# Mitochondrial Fatty Acid β-Oxidation Disorders: From Disease to Lipidomic Studies—A Critical Review

**DOI:** 10.3390/ijms232213933

**Published:** 2022-11-11

**Authors:** Inês M. S. Guerra, Helena B. Ferreira, Tânia Melo, Hugo Rocha, Sónia Moreira, Luísa Diogo, Maria Rosário Domingues, Ana S. P. Moreira

**Affiliations:** 1 Mass Spectrometry Center, LAQV-REQUIMTE, Department of Chemistry, University of Aveiro, Campus Universitário de Santiago, 3810-193 Aveiro, Portugal; 2 Centre for Environmental and Marine Studies—CESAM, Department of Chemistry, University of Aveiro, Campus Universitário de Santiago, 3810-193 Aveiro, Portugal; 3 Newborn Screening, Metabolism and Genetics Unit, Human Genetics Department, National Institute of Health Doutor Ricardo Jorge, 4000-053 Porto, Portugal; 4 Department of Pathological, Cytological and Thanatological Anatomy, School of Health, Polytechnic Institute of Porto, 4200-072 Porto, Portugal; 5 Internal Medicine, Centro Hospitalar e Universitário de Coimbra, 3000-075 Coimbra, Portugal; 6 Reference Center of Inherited Metabolic Diseases, Hospital Pediátrico, Centro Hospitalar e Universitário de Coimbra, 3000-075 Coimbra, Portugal

**Keywords:** inborn errors of metabolism, FAOD, MCADD, LCHADD, VLCADD, CPT2D, lipid changes, oxidative stress, lipidomics, mass spectrometry

## Abstract

Fatty acid oxidation disorders (FAODs) are inborn errors of metabolism (IEMs) caused by defects in the fatty acid (FA) mitochondrial β-oxidation. The most common FAODs are characterized by the accumulation of medium-chain FAs and long-chain (3-hydroxy) FAs (and their carnitine derivatives), respectively. These deregulations are associated with lipotoxicity which affects several organs and potentially leads to life-threatening complications and comorbidities. Changes in the lipidome have been associated with several diseases, including some IEMs. In FAODs, the alteration of acylcarnitines (CARs) and FA profiles have been reported in patients and animal models, but changes in polar and neutral lipid profile are still scarcely studied. In this review, we present the main findings on FA and CAR profile changes associated with FAOD pathogenesis, their correlation with oxidative damage, and the consequent disturbance of mitochondrial homeostasis. Moreover, alterations in polar and neutral lipid classes and lipid species identified so far and their possible role in FAODs are discussed. We highlight the need of mass-spectrometry-based lipidomic studies to understand (epi)lipidome remodelling in FAODs, thus allowing to elucidate the pathophysiology and the identification of possible biomarkers for disease prognosis and an evaluation of therapeutic efficacy.

## 1. Introduction

Mitochondrial fatty acid (FA) β-oxidation is a complex pathway which occurs in mitochondria to produce energy from lipids. The deficiency in specific transport proteins of FA or enzymes involved in FA β-oxidation is responsible for FA β-oxidation disorders (FAODs) [1]. More than 15 distinct disorders have been described, and the most prevalent FAODs are the medium-chain acyl-CoA dehydrogenase deficiency (MCADD), the long-chain hydroxy acyl-CoA dehydrogenase deficiency (LCHADD), and the very long chain acyl-CoA dehydrogenase deficiency (VLCADD) [2]. The clinical severity and age of onset are highly variable [1]. There is some overlap in the clinical phenotypes of diverse FAODs, such as acute hypoketotic hypoglycaemia and encephalopathy episodes. Although cardiac arrhythmias can occur in both MCADD and long-chain FAODs, cardiomyopathy and myopathy occur only in the latter [3]. Clinical phenotypes, with potential life-threatening manifestations, are the consequence of energy deficiencies and toxic effects of FA and acylcarnitine (CAR) accumulation [4,5]. To manage these diseases, patients need to follow a life-long avoidance of long fasting, a normal/low-fat diet, and carbohydrate and carnitine supplementation [1]. Both the disturbance of FAO and diet modifications can cause changes in the lipidome in FAOD patients. These can have a role in disease pathophysiology, determining clinical manifestations and prognosis.

Lipids have multiple functions in cells/biofluids, namely as an energy source, or as a major components of cell membranes and signalling molecules. Disturbances in the lipid profile have been associated with the pathophysiology of several diseases [6,7,8,9,10]. In FAODs, changes in lipids, namely in free and esterified FAs of plasma, dried blood spots (DBSs), and tissues have been reported. Despite minimal evidence that polar lipids, the main components of cell/organelle membranes and signalling molecules, can be affected in FAODs, the plasticity of the profile of these complex lipids has been scarcely studied [11,12].

Understanding the imbalance of the lipid profile can be useful in order to identify possible biomarkers for the monitoring of disease and therapeutic approaches. In this review, after a brief overview of mitochondrial FA β-oxidation (FAO), the state of the art of lipidomic analysis associated with FAODs is provided, with a perspective on its potential as a diagnostic and monitoring tool.

## 2. Mitochondrial Fatty Acid β-Oxidation

The mitochondrial FAO is one of the main pathways for energy production and is important for cell homeostasis [13,14,15,16]. FAO has a major role during metabolic stress, febrile illness, exercise, and fasting conditions, when the glucose levels in the blood decrease, providing an alternative fuel and sparing glucose [13,14,15,16].

**Figure 1:**
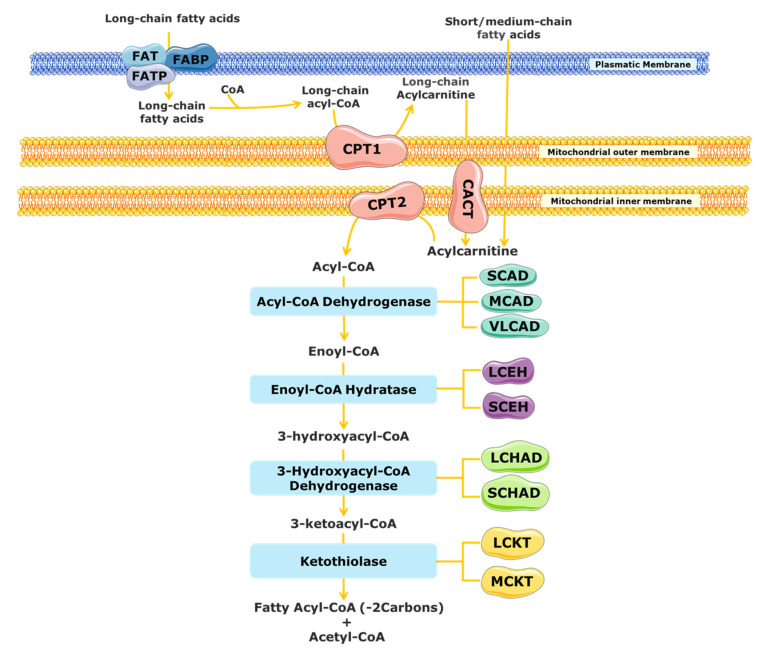
Schematic representation of mitochondrial fatty acid β-oxidation. CACT, carnitine acylcarnitine translocase; CPT, carnitine palmitoyltransferase; FABS, fatty-acid-binding protein; FAT, fatty acid translocase; FATP, fatty acid transport protein; LCHAD, long-chain 3-hydroxyacyl-CoA dehydrogenase; MCAD, medium-chain acyl-CoA dehydrogenase; SCAD, short-chain acyl-CoA dehydrogenase; SCHAD, short-chain 3-hydroxyacyl-CoA dehydrogenase; VLCAD, very long chain acyl-CoA dehydrogenases; LCEH, long-chain enoyl-CoA hydratase; SCEH, short-chain enoyl-CoA hydratase; LCKT, long-chain 3-ketoacyl-CoA thiolase; MCKT, medium-chain ketoacyl-CoA thiolase.

The substrates for mitochondrial FAO come from three main sources: (1) FAs from the diet or mobilized from other tissues that enter into the cell via the blood stream, (2) the de novo synthesis of FAs, and (3) FAs released within the cell through the hydrolysis of phospholipids (PLs) and triglycerides (TAGs) [17,18]. A schematic representation of mitochondrial FAO is shown in Figure 1. FAs from the diet and adipose tissue are transported through the blood stream bound to albumin and lipoproteins [19,20]. Upon reaching the target cells, the short- and medium-chain FAs cross the plasmatic membrane by passive diffusion [15]. In contrast, long-chain FAs are actively transported across the cell membrane by specific protein carriers present in the plasmatic membrane, namely FA transport proteins (FATPs), FA translocase (FAT), and FA-binding proteins (FABPs) (Figure 1) [13,15,19]. Once in the cytosol, long-chain FAs are converted into acyl-CoA esters, while short- and medium-chain FAs cross the mitochondrial membrane by passive diffusion and are converted to acyl-CoA esters inside the mitochondrial matrix [15,20,21]. As the mitochondrial membrane is not permeable to long acyl-CoA esters, the carnitine shuttle is required [13,19,22]. It needs the action of three distinct membrane-bound proteins: the carnitine palmitoyl transferase 1 (CPT1), which catalyses the formation of acylcarnitines (CARs) from acyl-CoA esters and free l-carnitine; the mitochondrial carnitine–acylcarnitine translocase (CACT), responsible for the exchange of a CAR for a free l-carnitine molecule from the mitochondrial matrix; and the carnitine palmitoyl transferase 2 (CPT2), which re-esterifies the CAR to the respective acyl-CoA esters [13,15,16,19,23]. Inside the mitochondrial matrix, the activated form of the FA (fatty acyl-CoA) is degraded by β-oxidation into acetyl-CoA units through a cyclic process.

Each β-oxidation cycle involves four enzymatic steps, which results in the release of two-carbon units (as acetyl-CoA) from the FA, with formation of a shorter FA [13]. These steps are catalysed by several enzymes with chain-length specificities, which overlap [15,21]. The first step, the dehydrogenation of the acyl-CoA ester to a trans-2-enoyl-CoA, is catalysed by different acyl-CoA dehydrogenases [15,24]. The short-chain acyl-CoA dehydrogenase (SCAD) acts on substrates with a chain length between C4 and C6, the medium-chain acyl-CoA dehydrogenase (MCAD) acts on C6 to C12 substrates, and the very long chain acyl-CoA dehydrogenase (VLCAD) is active with substrates ranging from C14 to C24 [16,23,25]. Following the initial dehydrogenation, a hydrogenation step of trans-2-enoyl-CoA is catalysed by 2-enoyl-CoA hydratases, producing an l-3-hydroxyacyl-CoA. At least two enzymes can carry out this reaction, the long-chain enoyl-CoA hydratase (LCEH) and the short-chain enoyl-CoA hydratase (SCEH), which are responsible for the hydration of long- and medium/short-chain enoyl-CoA species, respectively [14,23,25,26]. In the third step of the β-oxidation cycle, the resulting l-3-hydroxyacyl-CoA is dehydrogenated to 3-keto-acyl-CoA by a 3-hydroxyacyl-CoA dehydrogenase. The medium/short-chain 3-hydroxyacyl-CoA dehydrogenase (M/SCHAD) is responsible for the reduction of the medium- and short-chain l-3-hydroxyacyl-CoA. The long-chain 3-hydroxyacyl-CoA dehydrogenase (LCHAD), located at the α-subunit of the mitochondrial trifunctional protein (MTP), is responsible for the reduction of long-chain substrates [14,25,26]. The final step of the FAO cycle is represented by a thiolytic cleavage of the l-3-hydroxyacyl-CoA to yield an acetyl-CoA (or a propionyl-CoA, in the case of 2-methyl-branched-chain FA) and a two-carbon short-chained fatty acyl-CoA. This step is catalysed by the medium-chain 3-ketoacyl-CoA thiolase (MCKT), which acts on substrates between C4 and C12. The long-chain 3-ketoacyl-CoA thiolase (LCKT) is long-chain-specific [14,25,26]. Similar to the LCHAD enzyme, LCEH and LCKT are also part of the MTP [2].

Overall, there are at least twenty-five enzymes and specific transport proteins involved in mitochondrial FAO. Defects in many of them are associated with human diseases, notably FAODs, as will be detailed in the next section.

## 3. Mitochondrial Fatty Acid β-Oxidation Disorders (FAODs)

FAODs are autosomal recessive genetic defects and represent an important group of inborn errors of metabolism (IEMs). Although individually rare, they are relatively common as a group [16,27]. The most common FAODs can be divided in two groups, depending on the length of the FA affected: medium- and long-chain FAODs [26,28]. The former is MCADD and it is the most prevalent FAOD. The latter group includes several disorders of long-chain dehydrogenases and the FA mitochondrial carnitine transport system. LCHADD and VLCADD are the most prevalent of all long-chain FAODs [27,29]. FAODs have distinct clinical presentations and therapeutic needs [27]. Symptoms are triggered by long fasting, exercise, fever, and other causes of metabolic stress, such as surgery or trauma. Clinical presentation and severity are highly variable and may include acute hypoketotic hypoglycemia, peripherical neuropathy, cardiomyopathy, arrhythmias, hepatopathy, and/or myopathy [3,30].

FAODs are characterized by the accumulation of CARs, namely hexanoylcarnitine (CAR 6:0), octanoylcarnitine (CAR 8:0), decanoylcarnitine (CAR 10:0), and decenoylcarnitine (CAR 10:1 *n*-6, in MCADD [16,31]; 3-hydroxylcarnitines, namely 3-hydroxyhexadecanoylcarnitine (CAR 16:0;O), 3-hydroxyhexadecenoylcarnitine (CAR 16:1;O), 3-hydroxyoctadecanoylcarnitine (CAR 18:0;O), and 3-hydroxyoctadecenoylcarnitine (CAR 18:1;O) in LCHADD [16,27]; and tetradecenoylcarnitine (CAR 14:1) (the primary marker of this condition), tetradecanoylcarnitine (CAR 14:0), tetradecadienylcarnitine (CAR 14:2), dodecenoylcarnitine (CAR 12:1), and hexadecanoylcarnitine (CAR 16:0) in VLCADD [16,32]. The accumulation of CARs, as well as the respective free FAs, causes lipotoxicity and alters cell homeostasis [4,27].

FAODs can have life-threatening manifestations, namely hypoketotic hypoglycemia, encephalopathy, cardiac arrythmias and cardiomyopathy with heart failure, and rhabdomyolysis crisis with acute renal failure. Early diagnosis and treatment can be effective to prevent most of those complications, reducing morbidity and mortality. Therefore, FAODs are included in the newborn screening (NBS) program of several European countries [7,33]. The NBS of FAODs is based on the quantification of specific CARs in dried blood spots (DBSs) using highly sensitive techniques based on tandem mass spectrometry (MS/MS) for targeted analysis [2,34,35]. In addition to the identification of specific CAR profiles, CAR ratios are also analysed. For example, in VLCADD, the use of the CAR 14:1/CAR 2:0 and CAR 14:1/CAR 12:1 ratio helps to reduce the risk of false-negative results and improve the sensitivity of NBS diagnoses [4,15,36]. Afterwards, a definitive test by enzymatic and/or genetic analysis is performed to confirm the diagnosis [37]. In Table 1, the incidence, clinical presentation, and the characteristic CAR profile used in NBS for some FAODs are presented.

In general, the therapeutic management of FAODs aims to balance the energy deprivation and accumulation of toxic intermediates resultant from these metabolic defects [37]. MCADD patients can usually be managed with avoiding long fasting and with carbohydrate and carnitine supplementation, as needed [38,39]. In long-chain FAODs, a lipid-restricted diet is needed, with an important part of the usual dietary lipids (long-chain) substituted by medium-chain triglycerides containing C8 FAs (MCT-C8). The nutritional guidelines for FAOD therapy differ, depending on the FAOD subtype and the symptomatic/asymptomatic status of the patient [29]. Other therapeutic approaches, such as the use of anaplerotic therapy (triheptanoin) and bezafibrates, can be used in selected cases. Triheptanoin is a triglyceride composed by three odd-chain C7 FAs (MCT-C7) and can be used also as an alternative to MCT-C8 supplementation [31,36,37]. The advantage of using MCT-C7 instead of MCT-C8 is characterized by an anaplerotic effect by propionyl-CoA which can be fed into the citric acid cycle and produce succinyl-CoA, succinate, fumarate, malate, and oxaloacetate, which allows gluconeogenesis [31,36,37]. The bezafibrates represent a group of peroxisome proliferator-activated receptor (PPAR) agonists that are able to reduce lipid levels in human blood [36]. However, more clinical studies are needed to evaluate the effectiveness of these alternative therapeutics. Meanwhile, the dietary approach is the cornerstone for lifelong treatment in many FAODs.

As mentioned above, the inefficient use of FAs as an energy source and the accumulation of specific CARs and FAs in FAODs, as well as the restriction of essential FAs and other lipids in the diet and the MCT supplementation, can alter lipid homeostasis. Indeed, alterations in the lipid diet have been related to changes in the FA profile and membrane lipids, impacting the lipid metabolism and cellular function [40,41]. Moreover, the accumulation of specific CARs and FAs can reduce the FA intake by the cell, increase oxidative stress, and cause oxidative damage to lipids, changing the cellular lipid profile [4]. These changes may be involved in pathophysiology FAODs, as will be detailed in the next section.

**Table 1:** Some of the defects of the mitochondrial fatty acid β-oxidation with worldwide and Portuguese prevalence, clinical presentations, and newborn screening (NBS) acylcarnitine profile. Adapted from Sim et al. [15], Knottnerus et al. [2], Merritt et al. [31], and El-Gharbaway et al. [42].

Deficiency	Disorder Abbreviation	Worldwide Prevalence (Portugal in 2020)	Hypoketotic Hypoglycemia	Rhabdomyolysis	Cardiomyopathy	Skeletal Myopathy	Liver Dysfunction	Encephalopathy	Peripherical Neuropathy	Retinopathy	Acylcarnitine Marker (NBS)
**Mitochondrial β-oxidation spiral**											
Medium-chain acyl-CoA dehydrogenase	MCADD	1:4000 to 1:15,000 (1:7005)	X	-	-	-	X	X	-	-	CAR8:0, CAR8:0/CAR10:0 and CAR8:0/CAR2:0
Very long chain acyl-CoA dehydrogenase	VLCADD	1:30,000 to 1:100,000 (1:129,272)	X	X	X	X	X	-	-	-	CAR 14:1, CAR 14:2, CAR 14:1/CAR 2:0 and CAR 14:1/CAR 12:1
Long-chain 3-hydroxyacyl-CoA dehydrogenase	LCHADD	1:110,000 to 1:150,000 (1:94,800) *	X	X	X	X	X	-	X	X	CAR 16:0;O, CAR 18:1;O and CAR 18:0;O
**Carnitine shuttle**											
Carnitine palmitoyl transferase deficiency type 1	CPT1D	1:500,000 (1:473,998)	X	-	-	-	X	-	-	-	Free carnitine/(CAR16:0 + CAR18:0)
Carnitine palmitoyl transferase deficiency type 2	CPT2D	Rare (1:284,399)	X	X	X	X	X	-	-	-	(CAR16:0 + CAR18:0)/CAR2:0
Carnitine–acylcarnitine translocase	CACTD	Rare (1:284,399)	X		X	X	X	-	-	-	(CAR16:0 + CAR18:0)/CAR2:0

* This prevalence is referred to general MTP deficiencies (not differentiated based on the CAR profile in the NBS). Isolated LCHAD deficiency is the most common disorder of this complex [42].

## 4. Oxidative Stress and Lipids in FAOD Pathogenesis

**Figure 2:**
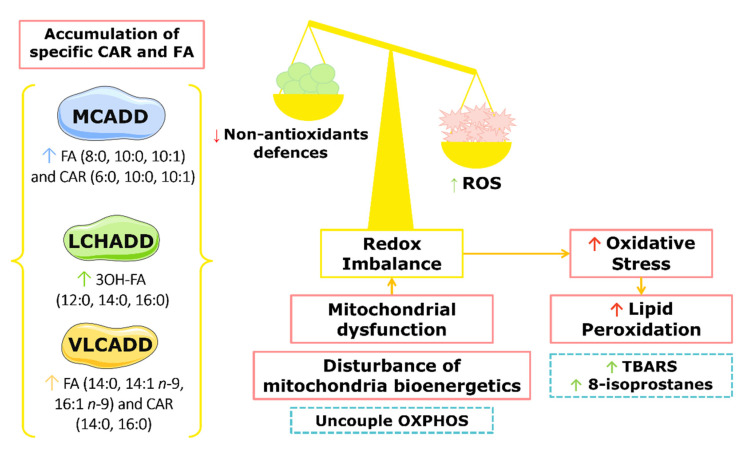
Schematic representation of the effects of specific FA and CAR accumulation in the pathogenesis of FAODs, its correlation with oxidative damage, and consequent disturbance of mitochondrial homeostasis.

FAODs affect the function of several organs, including heart, liver, skeletal muscle, and brain. Decrease in the energy production is proposed as one of the main alterations occurring in FAODs, but FAOD pathophysiology is not yet fully understood. Other mechanisms have been suggested, such as disturbed mitochondrial homeostasis, oxidative stress with increased production of reactive oxygen species (ROS), and the accumulation of toxic lipids (such as FAs and CARs) and oxidized lipids (such as oxidized phospholipids (PLs)) (Figure 2) [27,28,43,44].

The effect of the accumulation of medium-chain FAs and CARs (such as 8:0, 10:0, and 10:1) that occur in MCADD on oxidative stress parameters was investigated in cerebral cortex and skeletal muscle rat tissues [45,46,47,48]. When some of these FAs and CARs were included in the tissues’ incubation media, a reduction in non-enzymatic antioxidant defences, such as reduced glutathione (GSH), occurred, indicating an increase in the oxidative stress level [45,46,47,48]. Moreover, the accumulation of FAs 8:0, 10:0, and 10:1 led to mitochondrial dysfunction and bioenergetic impairment in MCADD rat tissues [48,49,50]. In the liver, FAs 10:0 and 10:1 uncoupled the oxidative phosphorylation (OXPHOS), leading to disruptions in the redox homeostasis of mitochondria and energy deficiency, but medium-chain CARs have not been shown to cause any changes in the mitochondrial homeostasis [49]. Still, they may induce oxidative stress in the cerebral cortex of young rats, possibly contributing to the neurological symptoms of MCADD [46].

The oxidative damage of biomolecules induced by the accumulation of medium-chain FAs and CARs in MCADD was investigated, and increases in the sulfhydryl oxidation or the formation of carbonyl derivatives (markers for assessing the protein oxidation) were found in skeletal muscle, liver, and the cerebral cortex of rats [45,46,47,48]. In the skeletal muscle and liver from rats, it was also observed that the incubation of FAs 8:0 and 10:0 markedly increased the production of thiobarbituric-acid-reactive substances (TBARSs) and spontaneous chemiluminescence, giving evidence of lipid oxidative damage [45,46,47].

The mitochondrial dysfunction could also play an important role in the pathophysiology of LCHADD [37], as supported by several studies showing that the accumulation of 3-hydroxy FAs (such as 12:0;O, 14:0;O, and 16:0;O) markedly disturbs the energy and redox homeostasis [51,52,53,54,55,56,57]. Moreover, the occurrence of lipid peroxidation and protein oxidative damage was corroborated by the high levels of TBARSs and by the increased carbonyl formation observed after the incubation of rat cerebral cortex tissue with 3OH-FA 12:0;O, 14:0;O, and 16:0;O [58]. The incubation with two of these 3OH-FA (14:0;O and 16:0;O) induced a decrease in GSH levels, showing a reduction in the brain non-enzymatic antioxidant defences [58]. Despite the evidence obtained from rat tissues, oxidative stress induced by the accumulation of the 3OH-FA was scarcely studied in LCHADD patients. One study showed increased levels of 8-isoprostanes, nitrite, and nitrate species in the urine of LCHADD patients, suggesting that lipid and DNA oxidative damage occurred due to an increase in reactive nitrogen and oxygen species (RNS and ROS) [59].

In VLCADD, studies with rats have shown that the accumulation of FAs 14:1 *n*-9 and 14:0 led to the disruption of mitochondrial functions, namely the uncoupled OXPHOS and the inhibited activity of the respiratory chain complexes I–III [60]. Furthermore, the incubation with FAs 14:1 *n*-9 and 14:0 induced similar effects in mitochondria from heart and skeletal muscle [61,62]. The monounsaturated FAs 14:1 *n*-9 and 16:1 *n*-9 induced an increase in the expression of the toll-like receptor 4 (TLR-4) and a decrease in the mitochondrial membrane potential, thus inducing apoptosis and necrosis in murine HL-1 cardiomyocytes [63]. These results support the hypothesis that the accumulation of these FAs may have a role in the apoptosis of cardiomyocytes and consequent cardiomyopathy in VLCADD. On the other hand, the toxicity of long-chain CARs was also associated with the pathogenesis of VLCADD [64].

In fibroblasts from VLCADD patients, a significant bioenergetic impairment with increased superoxide production, decreased mitochondrial respiratory chain function and low ATP levels were reported [65]. Furthermore, these abnormalities were correlated with an imbalance in ROS levels, and reduced mitochondrial respiration [65,66]. Moreover, increased levels of Nrf2 and NF-kB nuclear proteins were observed in VLCADD fibroblasts, suggesting the activation of signalling pathways involved with redox-state and inflammatory processes [65]. Taken together, the available studies in VLCADD strongly suggest that FA and CAR accumulation can act as uncouplers of OXPHOS, leading to metabolic inhibition and cell death.

## 5. Deregulation of the Lipidome in FAODs

Lipids are important key biomolecules in human physiology. The deregulation of lipid metabolisms and profiles has been associated with the pathophysiology of several diseases [67,68,69]. An analysis of the specific deviation of the lipidome, in a disease state, may allow the identification of biomarkers, either for diagnosis or prognosis, but also for disease monitoring and an evaluation of therapeutic outcomes [70,71].

Studies on alterations in fatty acids (FAs), acylcarnitines (CARs), and complex lipids in FAODs were gathered in this review. A search of the Scopus database was conducted with the following keywords: lipid profile, lipid change(s), lipidomic(s), lipidomic(s) profiling, acylcarnitine(s), fatty acid(s), medium-chain fatty acids, long-chain fatty acids, complex lipid(s), phospholipid(s), sphingomyelin, sphingolipid(s), ceramide(s), cardiolipin(s), lysophosphatidylcholine(s), and oxidized, combined with FAODs, MCADD, LCHADD, VLCADD, CPT2D, long-chain fatty acid oxidation disorders, and MCTs. All studies published until 2022 were analysed. Only research studies that used gas chromatography coupled with mass spectrometry (GC–MS), gas chromatography–flame ionization detection (GC-FID), liquid chromatography coupled with mass spectrometry (LC–MS), and direct injection mass spectrometry (DI-MS) were considered. A total of 15 published works (Supplementary Table S1) met the selection criteria and described alterations in the lipid profile of humans and mice with different FAOD. The main matrices used to evaluate those alterations were plasma, red blood cells (RBCs), DBSs, tissues, and fibroblasts. The results from these different works were stratified into four sub-sections and summarized in Table 2, Table 3, Table 4 and Table 5, where the alterations in FAs, CARs, and complex lipids (such as phospholipids (PLs), sphingolipids, and glycerolipids) are described according to the type of FAOD: MCADD, LCHADD, VLCADD, and CPT2D.

### 5.1. Changes in Fatty Acids, Acylcarnitines, and Complex Lipid Profiles in MCADD

Changes in FA profiles have been reported in MCADD. The increased levels of free FAs (FFAs), i.e., 8:0, 10:0, and 10:1 *n*-6, were identified in the plasma of six MCADD patients (Table 2) by GC-MS [72]. High plasma levels of the later FFA, as well as the 6:0, 12:0, 12:1, 14:0, 14:1 *n*-9, 14:2, 16:0, 16:1, 16:2, 18:0, 18:1, and 18:2, when compared to the established reference values, were reported in one MCADD patient [73]. In the same study, increased levels of 3-hydroxyl derivatives (6:0;O and 8:0;O) were also found [73]. The increase in those derivatives occurred in all FAOD cases evaluated in the study, suggesting that the origin of these is probably not related to a specific genetic disturbance of FAODs [73]. In DBS samples from two MCADD patients, the FFAs 8:0, 10:0, and 10:1 *n*-6 were markedly increased as well, when compared with controls [74], which is in agreement with previous studies on plasma FA profiles [72,73].

The total FA (TFA) composition was determined in *post-mortem* liver tissue from one MCADD patient [75]. Increased amounts of FAs 10:1 *n*-6, 12:1, 14:1 *n*-9, 14:2, and 16:2 *n*-6 (present as free and/or esterified FA) were found in the total lipid extract [75]. The increased levels of 10:1 *n*-6, either in DBSs or *post-mortem* liver tissue, were due to the partial β-oxidation of the linoleic acid (18:2 *n*-6), as the enzymes VLCAD and LCAD were active in MCADD [74,75]. After the total lipid extract was fractionated, it was found that all those FAs were only present in the triglyceride (TAG) fraction and were not detected in FFA or PL fractions. Thus, the authors hypothesized that these FAs (10:1 *n*-6, 12:1, 14:1, 14:2, and 16:2 *n*-6) originated from partial β-oxidation of mono- and polyunsaturated FAs, either in the mitochondria or in the peroxisomes [75]. They presumed that these FAs could migrate from these two organelles to the cytosol, in the form of CARs, and then be esterified into TAGs. The fact that these FAs were not incorporated in PLs may prevent a potential destabilizing effect on the membranes [75].

Regarding the complex lipid profile, only one study reported changes in MCADD, specifically in the DBSs of patients by using an untargeted MS-based metabolomic approach [44]. In addition to the increased levels of CAR 6:0, CAR 8:0, CAR 10:0, and CAR 10:1 *n*-6 (known as diagnosis markers of MCADD), three oxidized phosphatidylcholines (PCs), i.e., PC (16:0/9:0(COOH)), PC (18:0/5:0(COOH)), and PC (16:0/8:0(COOH)), were found. The oxidized PC may be correlated with the increased oxidative stress reported in MCADD patients (Section 4), which was also corroborated by the high levels of urinary 8-isoprostanes, as other lipid oxidation products increased under oxidative stress conditions [59].

In summary, changes in the lipid profile (of FAs, CARs, and complex lipids) were identified in MCADD, in the blood, plasma/serum, and DBSs, as well as in *post-mortem* liver tissue. Such changes can be due to an increase in the oxidative stress and/or the adaptation of lipid metabolism. Despite the fact that no studies related oxidation in PC species with pathophysiological mechanisms of MCADD, it cannot be excluded that they play a role in the development of comorbidities. For example, oxidized phospholipids have been associated with the development of cardiovascular disease and inflammation [76,77].

**Table 2:** Main lipid changes in plasma, *post-mortem* liver tissue, and dried blood spots (DBSs) of MCADD patients reported in published studies.

MCADD			
Sample	Decreased	Increased	Reference
Plasma	-	FFAs (8:0, 10:0, 10:1 *n*-6)	[72]
	-	FFAs (6:0, 8:0, 10:0, 10:1 *n*-6, 12:0, 12:1 14:0, 14:1 *n*-9, 14:2, 16:0, 16:1, 16:2, 18:0, 18:1, and 18:2); 3OH-FAs (6:0;O and 8:0;O)	[73]
DBS	-	FFAs (8:0, 10:0, and 10:1 *n*-6)	[74]
	-	CARs (6:0, 8:0, 10:0, and 10:1); PCs (16:0/9:0(COOH), 18:0/5:0(COOH), and 16:0/8:0(COOH))	[44]
*Post-mortem* liver tissue	-	TFAs (10:1 *n*-6, 12:1, 14:1, 14:2, and 16:2 *n*-6)	[75]

FFA(s), free fatty acid(s); 3OH-FA(s), 3-hydroxy fatty acid(s); DBS(s), dried blood spot(s); CAR(s), acylcarnitine(s); PC(s), phosphatidylcholine(s); TFA(s), total fatty acid(s).

### 5.2. Changes in Fatty Acids, Acylcarnitines, and Complex Lipid Profiles in LCHADD

An analysis of the plasma samples of three LCHADD patients revealed high levels of FFAs 14:1 *n*-9, 14:2, and 16:1 *n*-6 when compared with reference values (Table 3) [72]. On the other hand, decreased 18:2 *n*-6 levels (free and esterified) were found in the plasma and RBCs from LCHADD patients, treated with a low-fat diet and adequate essential FAs [78]. The arachidonic acid (AA, 20:4 *n*-6) level was within the reference range, despite the low level of its precursor (18:2 *n*-6) [78]. The levels of 3OH-FAs with a chain length ranging from C6 to C18 were also found to be increased in the plasma of LCHADD patients, when compared with reference levels [73]. The influence of diet on the CARs and 3-hydroxyacylcarnitine profiles in the plasma and DBSs of LCHADD patients under a low-fat diet was studied [79]. High levels of long-chain CARs (14:0, 14:1, 16:0 and 18:1) and their respective 3-hydroxyacylcarnitines, as well as of CAR 14:2;O, were found in both plasma and DBS samples [79]. CAR 18:2 was elevated only in plasma samples [79].

**Table 3:** Main changes in the lipid profile of plasma, red blood cells, dried blood spots (DBSs), and skin fibroblasts of long-chain hydroxy acyl-CoA dehydrogenase deficiency (LCHADD) patients reported in published studies.

LCHADD				
Sample	Additional Information	Decreased	Increased	Reference
Plasma		-	FFAs (14:1 *n*-9, 14:2, and 16:1)	[72]
		-	3OH-FAs (6:0;O, 8:0;O, 10:0;O, 12:0;O, 14:0;O, 14:1;O, 14:2;O, 16:0;O, 16:1;O, 16:2;O, 18:0;O, 18:1;O, and 18:2;O)	[73]
		TFA 18:2 *n*-6	-	[78]
		-	CAR 12:0, CAR 14:0, CAR 14:1, CAR 14:2, CAR 16:0, CAR 18:1, CAR 18:2, CAR 14:0;O, CAR 14:1;O, CAR 16:0;O, and CAR18:1;O	[79]
	LCHADD and CPT2D (considered as one study group) vs. healthy controls	HDL-C, PC 33:2, PC 34:0;O, PC 34:1;O, PC 34:3;O, PC 35:1, PC 35:2, PC 35:3, PC 36:2;O, PC(P-34:2), SM (d18:1/14:0), SM (d18:1/21:0), SM (d18:2/23:0), SM 33:1, SM 38:1, SM 40:2, SM 41:1, SM 43:1, SM 43:2, Cer (d18:1/23:0), Cer (40:1), Cer(42:1) and PE 36:3;O	TAG (14:0/14:0/14:0), TAG 44:1, TAG 46:2, TAG 46:3, TAG 56:6, TAG 58:9, and PC 40:5	[80]
Red blood cells		TFA 18:2 *n*-6		[78]
DBS			CAR 14:0, CAR 14:1, CAR 14:2, CAR 16:0, CAR 18:1, CAR 14:0;O, CAR 14:1;O, CAR 16:0;O, and CAR 18:1;O	[79]
Human skin fibroblasts	Incubated with l-carnitine	Medium: CAR 5:0	Cells: CAR 16:0, CAR 18:1, CAR 16:0;O, and CAR 18:1;O	[81]
	Incubated with l-carnitine and palmitic acid	Medium: CAR 2:0, CAR 4:0, CAR 5:0, CAR 6:0, CAR 8:0 and CAR 10:0	Medium and cells: CAR 16:0 and CAR 16:0;O	
	Incubated with l-carnitine and linoleic acid	Medium: CAR 2:0, CAR 4:0, CAR 5:0 and CAR 10:1	Medium: CAR 14:2, CAR 18:2, and CAR 18:2;O Cells: CAR18:2;O	
	Investigate specific alterations (without detailed identification of lipid species)	PE, SM, PC and PE with C30 and C37	PC; PC-O; CL; DAG; TAG; HexCer; PC/PE ratio; PC with C32 and C34; PC-O with C37; PE with C34, C36, and C38; LysoPL with C18; CL with C66 and C70; CL 64:3; and CL 64:4	[82]
	Investigate effects of the incubation with MCTs (C7 and C8)	Upon C7: PC Upon C8: PC, CL, HexCer, PC/PE ratio	Upon C7: CL, DAG, TAG, and Cer Upon C8: PE, DAG, TAG, SM, Cer, and CL with C68	[83]

FFA(s), free fatty acid(s); 3 OH-FA(s), 3-hydroxy fatty acid(s); TFA(s), total fatty acid(s); CAR(s), acylcarnitine(s); CPT2D, carnitine palmitoyl transferase type 2 deficiency; HDL-C, high-density lipoprotein cholesterol; PC(s), phosphatidylcholine(s); PC-O(s), alkyl-acyl phosphatidylcholine(s); SM(s), sphingomyelin(s); Cer(s), ceramides; PE, phosphatidylethanolamine; CL(s), cardiolipin(s); HexCer(s), hexosylceramide(s); DAG(s), diacylglyceride(s); TAG(s), triacylglyceride(s).

The accumulation of 3-hydroxyacylcarnitines and non-hydroxylated CARs in a whole-cell system was inferred by the analysis of the CAR profile in cultured fibroblasts, as it mirrors the accumulation of acyl-CoA esters [81]. For that, CARs and 3-hydroxyacylcarnitines in cells and media of LCHADD-cultured fibroblasts, before and after incubation with l-carnitine (either isolated or combined with a FA), were analysed by MS/MS on a triple quadrupole mass spectrometer [81]. The LCHADD fibroblasts, when incubated with l-carnitine (without adding FA), revealed significantly increased levels of CAR 16:0, CAR 18:1, CAR 16:0;O, and CAR 18:1;O, when compared with healthy controls [81]. However, this increase was not observed in the culture medium. After adding palmitic acid (FA 16:0) to the culture medium, the levels of CAR 16:0 and CAR 16:0;O were significantly increased in both cells and media [81]. When the LCHADD fibroblasts were incubated with FA 18:2 *n*-6, a significant increase in the corresponding 3-hydroxyacylcarnitine (CAR 18:2;O) was observed in both cells and media. Additionally, CAR 14:2 and CAR 18:2 were detected in significantly higher levels in the medium [81]. These results support the hypothesis that CARs can leave the mitochondria via the reverse action of carnitine–acylcarnitine translocase and subsequently cross the cell membrane and enter into the bloodstream. Furthermore, these findings gave evidence that the 3-hydroxyacylcarnitines remained more in the intracellular space when compared with the corresponding acylcarnitines [81]. In the same study, the LCHADD fibroblasts were incubated with the FAs usually found in the MCT supplements (10:0 and 8:0) and there was no accumulation of abnormal acylcarnitines levels, as expected [81]. Furthermore, the effect of MCT supplementation on specific 3-hydroxylacylcarnitines (CAR 16:0;O and CAR 18:1;O) was investigated by an analysis of plasma and DBS samples of LCHADD patients. A significant reduction in the levels of these two 3-hydroxyacylcarnitines was detected in the plasma [79]. Both studies corroborated that MCT supplementation is a beneficial treatment for this disorder. A reduction in the accumulation of FA metabolites during the MCT treatment could mirror the improvement of the clinical outcome, suggesting that lipidomic analysis is useful in therapeutic monitoring.

While the FA and CAR profiles in plasma have been well characterized, little is known regarding complex lipids, such as PC, SM, or TAG, in LCHADD. The plasma metabolites of LCHADD and CPT2D patients, both following a low-fat diet and MCT supplementation, were analysed together as a long-chain FAOD group and compared with a healthy control group [80]. Five TAG species containing fatty acyl chains with a sum of 44 carbons or more were significantly higher in the plasma of the LCHADD/CPT2D group when compared to the control group. This increase was related to the blockage of the enzymes associated with long-chain FA metabolism in LCHADD/CPT2D patients [80]. Significant alterations in PC were reported as well [80]. Notably, PC 40:5 was significantly increased, while the other ten PC species (of which five of them were oxidized PC species), with a total carbon chain length ranging between 33 and 36, showed significantly decreased levels [80]. The authors suggested that the increase in the lipid species with 40 carbons (TAGs and PCs) could mirror an improved elongation of long-chain FAs, somehow more incorporated into PC and TAG species [80]. The LCHADD/CPT2D patients also revealed a significant reduction in nine sphingomyelins (SMs) and three ceramides (Cers) [80], possibly be due to an impairment in the synthesis of these lipids with a reduction in the incorporation of SMs and Cers into plasmatic lipoproteins [80]. Although this study does not present the FA composition of decreased PCs, SMs, and Cers, some of these lipid species had odd long-chain FAs, considering the total number of carbons [80].

Disease-specific alterations in the cellular lipidome were also investigated in human fibroblasts from LCHADD patients through a shotgun lipidomic approach [82]. The PE content was significantly lower, while the PC content and the PC/PE ratio were significantly increased in this disorder, when compared with the healthy controls [82]. An increase in the PC/PE ratio was previously associated with the deregulation in mitochondrial biogenesis, i.e., the process of growth and division of pre-existing mitochondria, which has already been reported for this disorder [66,84]. One of the proposed pathophysiologic mechanisms related with a higher PC/PE ratio is the inhibition of the calcium transport activity of sarco/endoplasmic reticulum Ca^2+^- ATPase [85,86]. The elevation in the alkyl-acyl PC (PC-O) class was also observed in LCHADD fibroblasts and was associated with an increase in oxidative stress in this disorder [82]. PC-O is related with antioxidant effects due to the ability to scavenge oxygen radical species [87,88].

Reduced levels of SM and increased levels of hexosylceramide (HexCer) were found in LCHADD fibroblasts, which was related to a possible redirection of the SM-Cer pathway towards the production of HexCer species [82]. As higher levels of HexCer were previously associated with the development of neurodegenerative disease [89,90], it can be hypothesized that this increase may contribute to the development of peripheral neuropathy reported in LCHADD. LCHADD fibroblasts had an increase in the content of cardiolipins (CLs), a lipid class exclusively found in the inner mitochondrial membrane. The lipid species CL 64:3 and CL 64:4 were increased, which was associated with the incorporation of the non-metabolized FA C16 into CL species. As the α-subunit of the MTP is crucial for CL remodelling, the modification in CL composition induced by LCHADD/MTPD may contribute to the deregulation of mitochondrial dynamics [66]. In addition to the changes observed in polar lipids, a significant accumulation of storage lipids (TAGs and DAGs) was reported in LCHADD fibroblasts when compared with controls [82], in a similar trend as previously reported in LCHADD plasma [80].

Considering that the therapy of LCHADD includes MCT-C8 or MCT-C7 supplementation, LCHADD human fibroblasts were incubated with MCT-C8 or MCT-C7 to evaluate the effect of these supplementations on the complex lipid profile [83]. The PC content in LCHADD fibroblasts incubated with MCT-C8 or MCT-C7 was significantly decreased when compared with LCHADD fibroblasts without incubation (control). The PE content was only elevated in cells treated with MCT-C8, presenting a decreased PC/PE ratio compared to untreated cells. As reported above, the pathological mechanism of LCHADD is related with an increase in the PC/PE ratio [82]. A decrease in the PC/PE ratio in cells treated with MCT-C8 suggested that disease phenotypes can be counteracted by this type of supplement. However, possible changes in the cell PC/PE ratio induced by MCT-C8 supplementation could lead to a deterioration of the metabolic efficiency and impairment of the electron transfer chain, as reported for other pathologies, including metabolic diseases [91,92]. Additionally, LCHADD fibroblasts incubated with MCT-C8 revealed a significant decrease in the total content of CLs, whereas with the use of MCT-C7 increased it, when compared with control. Despite the fact that this has not been reported by the authors, a decrease in the total CL content caused by the MCT-C8 can be associated with oxidized CLs, as this shift could result from the unregulated oxidative stress present in LCHADD [93]. Additionally, in LCHADD fibroblasts incubated with MCT-C8, an increase in the CL species with 68 carbons was reported, while in the MCT-C7 treatment, the increase was insignificant. The unsaturation degree of the CLs was significantly affected by both MCT treatments, with an increment of species and a lower number of double bonds (between 0 and 6 double bonds). These changes in the CL lipid species can cause alterations in the mitochondrial membrane and subsequently impact the membrane dynamics [94,95]. However, MCT-C7 supplementation was the only one which appeared to induce a clear metabolic response, as corroborated by proteomics analysis. The authors suggested that changes in the CL profile induced by MCT-C7 supplementation may improve the mitochondrial metabolic efficiency and respiration and reduce the likely compensatory up-regulation of OXPHOS components [83]. Moreover, changes in Cer/HexCer and SM/HexCer ratios to normal levels were observed after the incubation of LCHADD cells with MCT-C7, which was related with the reversion of the SM biosynthesis flux, confirming the beneficial outcomes associated with this supplement [83]. This finding was also corroborated by proteomic analysis, showing an up-regulation in proteins/enzymes of the sphingolipid signalling pathway and Cer biosynthesis [83]. Thus, the application of MCT supplementation may result in an alteration in the composition of membrane complex lipids, namely in PCs, PEs, SMs, and Cers. Furthermore, it should be noted that this study did not compare the LCHADD fibroblasts after MCT-C7 and MCT-C8 incubation with fibroblasts from healthy controls, preventing any assessment of the MCT supplement’s ability to counteract the alterations associated with LCHADD pathophysiology, and how far the treatment can restore healthy lipid profiles.

### 5.3. Changes in Fatty Acids, Acylcarnitines, and Complex Lipid Profiles in VLCADD

VLCADD is usually diagnosed by the increased levels of the CAR 14:1, CAR 14:2, CAR 14:1/CAR 2:0, and CAR 14:1/CAR 12:1 ratio (Table 1). However, alterations in the FA profile were also reported. The GC-MS analysis of FFAs in the DBSs and plasma of VLCADD patients showed high levels of the long-chain FA 14:1 *n*-9, and the long-chain FAs 14:2 and 16:1 or 16:2 were only observed in plasma samples (Table 4) [72,73,74]. High levels of the same FA (14:1, 14:2, and 16:2 *n*-6), together with 10:1 *n*-6 and 12:1, were found by GC-FID analysis of the total fatty acids (TFAs), either in total lipid extracts or the respective TAG fractions, from *post-mortem* liver and skeletal muscle tissues [75]. As proposed by Onkehout et al. [75], the 14:1 *n*-9 could be derived from the mitochondrial FAO of 18:1 *n*-9 and the 14:2 could be derived from mitochondrial FAO of 18:2 *n*-6. However, it was suggested that peroxisomes could play a possible role in the β-oxidation process, as one study showed that peroxisomes were able to convert AA (20:4 *n*-6) into 14:2. Additionally, increases in the FFA 14:1 *n*-9/14:0 ratio and in the 3OH-FA (6:0;O and 8:0;O) were found by GC-MS analysis in the plasma of VLCADD patients [73].

Given the findings in FA changes, modifications in the complex lipids of VLCADD can be expected. The disease-specific alterations in the cellular lipidome were investigated through a lipidomic approach in the human fibroblasts of VLCADD patients [82]. The PL profile of the human fibroblasts revealed disease-specific differences when compared to the control group. Increased levels of PCs, alkyl-acyl PCs (PC-O), CLs, and TAGs, as well as decreased levels of alkyl-acyl PE (PE-O) (mainly PE-O with C36 and C38), were found in VLCADD fibroblasts [82]. A higher content in lyso-PL (mainly C14 lyso-PL) species was also found in VLCADD cell lines [82]. Considering that lyso-PLs are usually pro-inflammatory mediators, this is in agreement with previous evidence of an increase in the inflammatory response in human VLCADD fibroblasts [96]. Elevated levels of C14 FAs and CARs were also reported in the plasma, DBSs, and fibroblasts of VLCADD fibroblasts [72,73,74,97,98]. The increase in lyso-PL species with C14 acyl chains can be a consequence of the action of the phospholipase A2 (PLA_2_), an enzyme that cleaves the *sn*-2 ester linkage of PLs. The PLA_2_ activity is usually upregulated during inflammation, which is involved in the pathophysiology of VLCADD fibroblasts (see Section 4).

The long-chain triglyceride restriction diet, enriched with MCTs, is the mainstay of VLCADD treatment. Thus, the long-term effect of MCT-C8 or triheptanoin (MCT-C7) supplementation was analysed in plasma samples of VLCADD patients [99]. The samples of VLCADD patients on MCT-C8 or MCT-C7 supplementation revealed significantly higher levels of very long chain acylcarnitines (CAR 17:0, CAR 20:0, CAR 22:0, and CAR 24:1) than healthy controls, regardless of the type of supplement [99]. Increased levels of odd-chain LPEs, LPCs, PCs, and SMs were identified only in patients supplemented with MCT-C7 (but not in those supplemented with MCT-C8). The mechanism for the increase in these lipids remains unknown, but studies on VLCADD mice indicate that the excess of substrate of the MCT enters in the complex lipid synthesis, after the hydrolysis and chain elongation of the MCT fatty acids [97,100].

**Table 4:** Main changes in the lipid profile in the plasma, dried blood spots (DBSs), and skin fibroblasts of very long chain acyl-CoA dehydrogenase deficiency (VLCADD) patients and mice reported in published studies.

VLCADD					
Sample		Additional Information	Decreased	Increased	Reference
Human	Plasma		-	FFAs (14:1 *n*-9, 14:2, and 16:1)	[72]
			-	FFAs (14:1 *n*-9, 14:2, and 16:2) 3OH-FAs (6:0;O and 8:0;O)	[73]
		Patients with MCT (C7 or C8) supplementation vs. healthy controls	-	C8 or C7: CAR 17:0, CAR 20:0, CAR 22:0, and CAR 24:1 C7: LPE 17:0, LPC 15:0, PC 17:0/20:4, PC 17:0/22:6, PC 15:0/20:4, PC 15:0/22:6, PC 15:0/18:1, PC 17:0/16:1, PC 16:0/17:1, SM d18:2/23:1, SM d17:2/16:0, and SM d18:2/15:0	[99]
	*Post-mortem* liver and muscle tissue		-	TFAs (10:1 *n*-6, 12:1, 14:1 *n*-9, 14:2, and 16:2 *n*-6)	[75]
Mice	Liver tissue	MCT (C7 or C8) vs. LCT	C7 or C8: FAs (18:2 *n*-6, 18:3 *n*-3, 20:4 *n*-6, 20:5, 22:6 *n*-3 and PUFA)	C7 or C8: FAs (14:0, 16:1, 18:1 *n*-9, 18:2 *n*-6, 20:1, 20:2, 20:3 *n*-9, and SFA MUFA) and cholesterol C7: FAs (15:0, 17:1, 18:0, and 22:1) C8: FA 22:5 *n*-3	[97]
	Heart tissue		C7 or C8: FAs (18:2 *n*-6, 18:3 *n*-3 and PUFA) C8: 16:0	C7 or C8: FAs (18:0, 20:1, 20:3 *n*-9, 22:4, SFA, and MUFA) C7: FAs (16:1, 17:1, and 22:4) C8: FAs (20:3 *n*-6 and 22:5 *n*-6) and cholesterol	
Human	DBS		-	FFA 14:1	[74]
Mice		MCT-VLCAD^-/-^ vs. LCT-VLCAD^-/-^	Male MCT-VLCAD^-/-^: CAR 4:0 and CAR 18:2 Female MCT-VLCAD^-/-^: CAR 3:0, CAR 18:2, and CAR 16:0;O	Male MCT-VLCAD^-/-^: CAR 16:0 and CAR 16:1 Female MCT-VLCAD^-/-^: CAR 4:0	[98]
		MCT-VLCAD^-/-^ vs. MCT-WT	-	Male MCT-VLCAD^-/-^: CAR 16:0, CAR 16:1, and CAR 18:0 Female MCT-VLCAD^-/-^: CAR 4:0	
		LCT-VLCAD^-/-^ vs. LCT-WT	-	Male LCT-VLCAD^-/-^: CAR 4:0 and CAR 16:0 Female LCT-VLCAD^-/-^: CAR 18:0	
Human	Fibroblasts	VLCADD vs. healthy controls	PE-O, PE-O with C36 and C38	PC, PC-O, CL, TAG, LysoPL, and LysoPL with C14	[82]
		Investigate effects of the incubation with MCT (C7 or C8)	C7: PE C8: PC and PE	C7: SM, HexCer, DAG, TAG, and PC/PE ratio C8: SM, HexCer, DAG, TAG, and CL with C66	[83]
		WT vs. VLCAD	Male VLCAD: PE, PI, and SM Female VLCAD: PI	Male VLCAD: PC-O, PE-O, and HexCer Female VLCAD: PC, PE-O, Cer, HexCer, and SM	[101]
		WT-C8 vs. VLCAD-C8	Male VLCAD-C8: PC, PE, PI, and PSFemale VLCAD-C8: PE, PI, and PS	Male VLCAD-C8: PC-O, PE-O, and HexCer Female VLCAD-C8: PC, PG, Cer, HexCer, and SM	
		VLCAD vs. VLCAD-C8	Male VLCAD-C8: PC and PE	Male VLCAD-C8: PC-O, and PE-OFemale VLCAD-C8: PC, LPC 16:0, LPC 16:1, LPC 18:0, and LPC 18:1	

FFA(s), free fatty acid(s); 3 OH-FA(s), 3-hydroxy fatty acid(s); TFA(s), total fatty acid(s); CAR(s), acylcarnitine(s); LCT(s), long-chain triacylglyceride(s); MCT(s), medium-chain triacylglyceride(s); MCT-C7(s), medium-chain long-chain triacylglyceride(s) containing C7; MCT-C8(s), medium-chain long-chain triacylglyceride(s) containing C8; WT, wild type; MUFA(s), monounsaturated fatty acid(s); SFA(s), saturated fatty acid(s); PUFA(s), polyunsaturated fatty acid(s); PC(s), phosphatidylcholine(s); PC-O(s), alkyl-acyl phosphatidylcholine(s); LPC, lysophosphatidylcholine; LPE, lysophosphatidylethanolamine; SM(s), sphingomyelin(s); Cer(s), ceramide(s); PE, phosphatidylethanolamine; PE-O(s), alkyl-acyl phosphatidylethanolamine(s); CL(s), cardiolipin(s); HexCer(s), hexosylceramide(s); DAG(s), diacylglyceride(s); TAG(s), triacylglyceride(s).

Changes in complex lipids were identified in the human fibroblasts of VLCADD patients after incubation with MCT-C8 or MCT-C7 [83]. Significantly reduced levels of PCs and PEs were observed in VLCADD fibroblasts after incubation with MCT-C8 and incubation with MCT-C8 or MCT-C7, respectively [83]. As previously described, VLCADD fibroblasts presented elevated levels of PC species, when compared to healthy controls [82]. The reduction in PC observed after VLCADD fibroblast incubation with MCT-C8 suggests that this type of supplement can counteract the disease phenotype. In parallel, an increase in the molar ratio of PC/PE was observed after incubation with MCT-C7 [83]. Furthermore, an increase in DAG levels was identified after the incubation of VLCADD human fibroblasts with either MCT-C8 or MCT-C7 [83]. Considering that the parallel increase in DAG and PE species induces regions of negative stress curvature in cellular membranes, it was suggested that the concomitant decrease in PE observed after the incubation of VLCADD human fibroblasts with MCTs may have a protective effect in cellular membranes [83,92]. Additionally, the levels of TAG, as well as SM and HexCer, were increased after incubation with MCT-C7 or MCT-C8, when compared with control conditions (without MCTs). Moreover, VLCADD fibroblasts incubated with MCT-C8 revealed a significant increase in CLs with 66 carbons, not observed with MCT-C7. Finally, it is possible to conclude that the incubation of VLCADD fibroblasts with these two types of MCT altered the composition of membrane complex lipids.

The effect of MCT supplementation, on the FA profile was also studied in cardiac and hepatic tissues of VLCAD^-/-^ mice [97]. After one year of MCT supplementation (C8 or C7), increased levels of FAs such as 14:0, 16:1, 18:1 *n*-9, and 20:1 were found in VLCADD^-/-^ liver, supporting the hypothesis that only some medium-chain FAs are β-oxidized, while the rest were elongated and stored as long-chain FAs [97]. Additionally, reduced levels of FAs 18:2 *n*-6 and 18:3 *n*-3 were found in the liver and heart tissues of VLCAD^-/-^ mice fed with an MCT (C8 or C7) diet, when compared with a long-chain triglyceride (LCT) diet. As a result, the levels of DHA (22:6 *n*-3) and AA (20:4 *n*-6), formed from these two FAs, decreased, but only in the liver [97]. The FAs 15:0, 17:1, and 18:0 were elevated in the liver of VLCAD^-/-^ mice fed with an MCT-C7 diet. The authors suggested that the accumulation of the odd-chain FAs 15:0 and 17:1 was probably due to the incorporation of the propionyl-CoA derived from the β-oxidation of the triheptanoin. The increase in the FA 15:0 was not observed in the heart of VLCAD^-/-^ mice fed with an MCT-C7 diet and the increase in the FA 17:1 was less pronounced than that observed in the liver tissue. This might be associated with a higher anaplerotic effect to meet the higher energy demand of the heart [97]. In summary, the long-term supplementation of even- or odd-chain MCTs in the VLCAD^-/-^ mice model led to an increase in the monounsaturated FA (MUFA) levels and a decrease in the polyunsaturated FA (PUFA) levels. The FAs 17:1 *n*-9, 20:1 *n*-9, 20:3 *n*-9, and 22:1 *n*-9 detected in VLCAD^-/-^ mice fed with MCT-C8 and MCT-C7, but absent in mice under control conditions, may be raised from de novo synthesis and elongation processes [102].

The CAR profile of DBS samples from VLCAD^-/-^ mice revealed a sex-specific response to MCT-C8 supplementation [98]. An increase in the total CAR content was found in male mice, which was attributed to the higher muscle mass in males associated with the regulation of plasma carnitine and thereby acylcarnitines by sex hormones [103]. On the other hand, a decreased content in CAR 18:2 was found in both sexes of VLCAD^-/-^ mice under the MCT diet, when compared to the normal LCT diet [98].

The sex-specific effects of MCT-C8 on complex lipids were also investigated through lipidomic analysis in fibroblasts from wild-type (WT) and VLCAD^-/-^ mice of both sexes [101]. Considering the fibroblasts from male mice, a reduction in the PC content of VLCAD^-/-^ incubated with MCT-C8 (VLCAD^-/-^-C8), when compared with either VLCAD^-/-^ (without MCT-C8 incubation) and WT incubated with MCT-C8 (WT-C8), was observed. Contrarily, a significant increase in PC content was observed after the incubation with MCT-C8 in VLCAD^-/-^ female. The concentration of PE in female and male VLCAD^-/-^-C8 mice showed decreased levels when compared with WT-C8 mice of the same sex. MCT-C8 also induced a remarkable increase in PC-O and alkyl-acyl PE (PE-O) endogenous antioxidants in male VLCAD^-/-^-C8 mice. This effect did not appear in female VLCAD^-/-^-C8 mice, suggesting a possible sex-specific redirection of the biosynthesis pathway of alkyl-acyl species in these cells. Indeed, the up-regulation of the biosynthesis of these alkyl-acyl PL species in male VLCAD^-/-^-C8 mice fibroblasts was corroborated by an increase in the expression of three genes involved in their biosynthesis. Although not being reported by the authors, the elevated levels of these endogenous antioxidants may have a protective effect on oxidative stress, previously reported in VLCADD patients [4]. In the same study, significant levels of LPC 16:0, LPC 16:1, LPC 18:0, and LPC 18:1 were exclusively observed in fibroblasts from female VLCAD^-/-^-C8 mice. This increase in pro-inflammatory LPC species was related to the MCT-induced activation of the mechanistic target of rapamycin (mTOR), leading to the stimulation of pro-inflammatory pathways [104].

Regarding the levels of phosphatidylserines (PSs) and phosphatidylinositols (PIs), both classes were decreased in cells from male and female VLCAD^-/-^ mice incubated with MCT-C8. None of the previously published studies had found alterations in these two PL classes in VLCADD. Diminished levels of PS were reported in apoptotic cells [105,106]. Moreover, PIs are an important precursor of phosphoinositides (PIPs) and a major contributor to signal transduction pathways, so their decrease may cause changes in the secretory cascades and intracellular signalling pathways [107]. Briefly, this study in mice underlines that VLCADD treatment with MCTs may have an effect, not only on the de novo biosynthesis and elongation of FA, as reported previously [97,97,100], but also on the homeostasis of cellular complex lipids, such as PLs. Although MCT-C8 led to the change in specific complex lipids in both sexes, the results were completely distinct. Further clarification on the effect of MCT-C8 supplementation in the VLCADD lipid profile is needed.

In summary, the studies performed to date demonstrated that VLCADD not only affects FA degradation, but also causes alterations in the profile of complex lipids. The high content of alkyl-acyl species and lyso-PL strongly supports the theory of an inflammatory process in this disorder. Additionally, it is possible to conclude that the even- or odd-chain MCT supplementation may result in an alteration in the composition of membrane complex lipids, some of which are sex-specific. Overall, the lipidomic approaches used in these studies reveal alterations in the composition of membrane PLs, but further research is necessary to fully understand the biological and physiological implications of these findings and the relationship with disease pathogenesis.

### 5.4. Changes in Fatty Acids, Acylcarnitines, and Complex Lipid Profiles in CPT2D

In CPT2D, the FA/3OH-FA and complex lipid profiles were only analysed in three studies (Table 5) [73,80,82]. The FFA and 3OH-FA profiles were investigated in plasma samples of CPT2D patients, and increased levels of FFA (16:0 and 18:0) and 3OH-FA (6:0;O and 8:0;O) were disclosed [73]. These findings are in agreement with the higher levels of the corresponding CAR 16:0 and CAR 18:0, used as biomarkers for the NBSs of this disease [2]. Alterations in metabolites, including CARs and complex lipids, as well as in the plasma samples of CPT2D patients, were published, together with LCHADD patient results (described above in Section 5.2), but it was not possible to prove if there were specific changes to CPT2D [80]. Disease-specific alterations in the complex lipids were analysed through a lipidomics approach in human fibroblasts from CPT2D patients [82]. The PC content was significantly reduced in CPT2D cell lines when compared with the control group. In contrast, PE levels were significantly increased in the CPT2D group, resulting in a remarkably reduced PC/PE ratio when compared with controls. In CPT2D cell lines, a decrease in PE-O levels (suggesting a reduction in antioxidant defences) and an elevated content of the pro-inflammatory lyso-PL (predominantly with C18) were also identified. The levels of TAG and DAG species were increased in CPT2D cells when compared with the controls [82].

Despite the few published studies, it is noticeable that CPT2D induces alterations in the lipid metabolism, which may be associated with a pro-inflammatory phenotype and a decrease in antioxidant defences, probably due to increased oxidative stress. However, further research is needed to fully understand how disorders in the FA metabolism change the profile of PLs; the main components of cell membranes, organelles, and lipoproteins; as well as the long-term effects of MCT supplementation.

**Table 5:** Main changes in lipids in plasma and skin fibroblasts of carnitine palmitoyl transferase type 2 deficiency (CPT2D) patients reported in published studies ^a^.

CPT2D			
Sample	Decreased	Increased	Reference
Plasma	-	FFAs (16:0 and 18:0) 3OH-FAs (6:0;O and 8:0;O)	[73]
Human skin fibroblasts	PC, PE-O, PC/PE ratio, PC and PE with C30 and C37, and PE-O with C36 and C38	PE, DAG, TAG, LysoPL, PC with C32 and C34, PE with C34, C36 and C38, LysoPL with C18, and CL with C66 and C68	[82]

^a^ An additional study [80] was performed with the plasma of LCHADD and CPT2D patients (considered as one study group) (see Table 3). FFA(s), free fatty acid(s); 3 OH-FA(s), 3-hydroxy fatty acid(s); PL(s), phospholipid(s); PC(s), phosphatidylcholine(s); PC-O(s), alkyl-acyl phosphatidylcholine(s); PE, phosphatidylethanolamine; PE-O(s), alkyl-acyl phosphatidylethanolamine(s); CL(s), cardiolipin(s); DAG(s), diacylglyceride(s); TAG(s), triacylglyceride(s).

## 6. Concluding Remarks and Future of Lipidomics in FAODs

Lipids are known to have important biological functions which play fundamental roles in many signalling pathways that are associated with the maintenance of cell and tissue homeostasis. The evidence gathered, either with samples from humans or mice, showed that the impairment in enzymes/transporters involved in mitochondrial FAODs can alter the lipid profile of patients. In addition to FA and CAR accumulation, there are also alterations at the level of different PL classes. A higher content in the pro-inflammatory lyso-PLs, which can possibly contribute to an inflammatory state, is emphasized. PL oxidation can lead to alterations in the lipid profile, which may influence the function of cells/organs that, to work well, need to preserve lipid homeostasis. Indeed, changes in the main membrane lipids, PCs and PEs, were reported in some FAODs. Consequently, these changes can alter the membrane curvature and fluidity. Furthermore, a decrease in the CL content was reported, which can be associated with mitochondrial dysfunction. In FAODs, alterations in the sphingolipid metabolism were also reported. Increased levels of HexCer were found, which were previously associated with the development of neurodegenerative diseases and may possibly contribute to the development of comorbidities (such as peripheral neuropathy). Moreover, there is growing evidence that the major CARs and FAs accumulating in each FAOD may induce an increase in oxidative damage, disturbing mitochondrial homeostasis and possibly contributing to the pathogenesis of FAOD patients.

Despite evidence of changes in a few polar lipids over the last few years, a comprehensive analysis of the plasticity of the lipidome of FAOD patients is still missing to understand how the overall lipid profile is altered in each FAOD. Most of the published studies used a metabolomic approach, which have also detected alterations in some lipid species, such as PCs. Those that used a lipidomic approach only analysed some lipid classes, leaving out a considerable number of lipid species, including less abundant and signalling lipids with important roles. On the other hand, the few studies published were mainly carried out using cells (fibroblasts) and mouse models. Few studies were performed with human samples, such as plasma, and this could be important to see if the alterations found in cell and mouse models are also observed in FAOD patients. In addition, there are ongoing efforts to tackle the lack of current harmonization in analytical procedures. The use of different analytical approaches in lipidomic studies, such as different lipid extraction methods and analysis workflows, hinders the comparability of results. Thus, more standardization is required to allow to better understanding of the possible repercussions of lipidomic changes in vital organs and the likelihood of the appearance of comorbidities. To summarize, the identification of lipidome plasticity in FAODs may help possible biomarkers to be identified, which are essential in order to monitor disease evolution and the effect of therapeutical approaches, possibly representing a promising tool for precision medicine.

## Supplementary Materials

The following supporting information can be downloaded at: https://www.mdpi.com/article/10.3390/ijms232213933/s1.

## References

[1] Moczulski D., Majak I., Mamczur D. (2009). An Overview of Beta-Oxidation Disorders. Postep. Hig. Med. Dosw..

[2] Knottnerus S.J.G., Bleeker J.C., Wüst R.C.I., Ferdinandusse S., IJlst L., Wijburg F.A., Wanders R.J.A., Visser G., Houtkooper R.H. (2018). Disorders of Mitochondrial Long-Chain Fatty Acid Oxidation and the Carnitine Shuttle. Rev. Endocr. Metab. Disord..

[3] Spiekerkoetter U., Sun B., Zytkovicz T., Wanders R., Strauss A.W., Wendel U. (2003). MS/MS-Based Newborn and Family Screening Detects Asymptomatic Patients with Very-Long-Chain Acyl-CoA Dehydrogenase Deficiency. J. Pediatr..

[4] Ribas G.S., Vargas C.R. (2020). Evidence That Oxidative Disbalance and Mitochondrial Dysfunction Are Involved in the Pathophysiology of Fatty Acid Oxidation Disorders. Cell. Mol. Neurobiol..

[5] Ruiz-Sala P., Peña-Quintana L. (2021). Biochemical Markers for the Diagnosis of Mitochondrial Fatty Acid Oxidation Diseases. J. Clin. Med..

[6] Hyötyläinen T., Orešič M. (2015). Analytical Lipidomics in Metabolic and Clinical Research. Trends Endocrinol. Metab..

[7] Guerra I.M.S., Ferreira H.B., Neves B., Melo T., Diogo L.M., Domingues M.R., Moreira A.S.P. (2020). Lipids and Phenylketonuria: Current Evidences Pointed the Need for Lipidomics Studies. Arch. Biochem. Biophys..

[8] Zhao Y.-Y., Miao H., Cheng X.-L., Wei F. (2015). Lipidomics: Novel Insight into the Biochemical Mechanism of Lipid Metabolism and Dysregulation-Associated Disease. Chem.-Biol. Interact..

[9] Züllig T., Trötzmüller M., Köfeler H.C. (2020). Lipidomics from Sample Preparation to Data Analysis: A Primer. Anal. Bioanal. Chem..

[10] Herzog K., Pras-Raves M.L., Ferdinandusse S., Vervaart M.A.T., Luyf A.C.M., van Kampen A.H.C., Wanders R.J.A., Waterham H.R., Vaz F.M. (2018). Plasma Lipidomics as a Diagnostic Tool for Peroxisomal Disorders. J. Inherit. Metab. Dis..

[11] Ivanovová E., Piskláková B., Dobešová D., Kvasnička A., Friedecký D. (2021). Novel LC-MS Tools for Diagnosing Inborn Errors of Metabolism. Microchem. J..

[12] Ismail I.T., Showalter M.R., Fiehn O. (2019). Inborn Errors of Metabolism in the Era of Untargeted Metabolomics and Lipidomics. Metabolites.

[13] Houten S.M., Wanders R.J.A. (2010). A General Introduction to the Biochemistry of Mitochondrial Fatty Acid β-Oxidation. J. Inherit. Metab. Dis..

[14] Houten S.M., Violante S., Ventura F.V., Wanders R.J.A. (2016). The Biochemistry and Physiology of Mitochondrial Fatty Acid β-Oxidation and Its Genetic Disorders. Annu. Rev. Physiol..

[15] Sim K.G., Hammond J., Wilcken B. (2002). Strategies for the Diagnosis of Mitochondrial Fatty Acid β-Oxidation Disorders. Clin. Chim. Acta.

[16] Kompare M., Rizzo W.B. (2008). Mitochondrial Fatty-Acid Oxidation Disorders. Semin. Pediatr. Neurol..

[17] Cooper D.E., Young P.A., Klett E.L., Coleman R.A. (2015). Physiological Consequences of Compartmentalized Acyl-CoA Metabolism. J. Biol. Chem..

[18] Nsiah-Sefaa A., McKenzie M. (2016). Combined defects in oxidative phosphorylation and fatty acid β-oxidation in mitochondrial disease. Biosci. Rep..

[19] Longo N., Frigeni M., Pasquali M. (2016). Carnitine Transport and Fatty Acid Oxidation. Biochim. Biophys. Acta (BBA)-Mol. Cell Res..

[20] Kerner J., Hoppel C. (2000). Fatty Acid Import into Mitochondria. Biochim. Biophys. Acta (BBA)-Mol. Cell Biol. Lipids.

[21] Wanders R.J.A., Ruiter J.P.N., IJlst L., Waterham H.R., Houten S.M. (2010). The Enzymology of Mitochondrial Fatty Acid Beta-Oxidation and Its Application to Follow-up Analysis of Positive Neonatal Screening Results. J. Inherit. Metab. Dis..

[22] Adeva-Andany M.M., Carneiro-Freire N., Seco-Filgueira M., Fernández-Fernández C., Mouriño-Bayolo D. (2019). Mitochondrial β-Oxidation of Saturated Fatty Acids in Humans. Mitochondrion.

[23] Bartlett K., Eaton S. (2004). Mitochondrial β-Oxidation. Eur. J. Biochem..

[24] Lund M., Olsen R., Gregersen N. (2015). A Short Introduction to Acyl-CoA Dehydrogenases; Deficiencies and Novel Treatment Strategies. Expert Opin. Orphan Drugs.

[25] Wanders R.J., Vreken P., den Boer M.E., Wijburg F.A., van Gennip A.H., IJlst L. (1999). Disorders of Mitochondrial Fatty Acyl-CoA Beta-Oxidation. J. Inherit. Metab. Dis..

[26] Rinaldo P., Matern D., Bennett M.J. (2002). Fatty Acid Oxidation Disorders. Annu. Rev. Physiol..

[27] Wajner M., Amaral A.U. (2016). Mitochondrial Dysfunction in Fatty Acid Oxidation Disorders: Insights from Human and Animal Studies. Biosci. Rep..

[28] Olpin S.E. (2013). Pathophysiology of Fatty Acid Oxidation Disorders and Resultant Phenotypic Variability. J. Inherit. Metab. Dis..

[29] Merritt J.L., MacLeod E., Jurecka A., Hainline B. (2020). Clinical Manifestations and Management of Fatty Acid Oxidation Disorders. Rev. Endocr. Metab. Disord..

[30] Blau N., Duran M., Gibson K.M., Dionisi-Vici C. (2014). Physician’s Guide to the Diagnosis, Treatment, and Follow-Up of Inherited Metabolic Diseases.

[31] Merritt J.L., Norris M., Kanungo S. (2018). Fatty Acid Oxidation Disorders. Ann. Transl. Med..

[32] Rovelli V., Manzoni F., Viau K., Pasquali M., Longo N. (2019). Clinical and Biochemical Outcome of Patients with Very Long-Chain Acyl-CoA Dehydrogenase Deficiency. Mol. Genet. Metab..

[33] Loeber J.G., Platis D., Zetterström R.H., Almashanu S., Boemer F., Bonham J.R., Borde P., Brincat I., Cheillan D., Dekkers E. (2021). Neonatal Screening in Europe Revisited: An ISNS Perspective on the Current State and Developments since 2010. Int. J. Neonatal Screen..

[34] Lindner M., Hoffmann G.F., Matern D. (2010). Newborn Screening for Disorders of Fatty-Acid Oxidation: Experience and Recommendations from an Expert Meeting. J. Inherit. Metab. Dis..

[35] Vilarinho L., Rocha H., Sousa C., Marcão A., Fonseca H., Bogas M., Osório R.V. (2010). Four Years of Expanded Newborn Screening in Portugal with Tandem Mass Spectrometry. J. Inherit. Metab. Dis..

[36] Yamada K., Taketani T. (2019). Management and Diagnosis of Mitochondrial Fatty Acid Oxidation Disorders: Focus on Very-Long-Chain Acyl-CoA Dehydrogenase Deficiency. J. Hum. Genet..

[37] Wanders R.J.A., Visser G., Ferdinandusse S., Vaz F.M., Houtkooper R.H. (2020). Mitochondrial Fatty Acid Oxidation Disorders: Laboratory Diagnosis, Pathogenesis, and the Complicated Route to Treatment. J. Lipid Atheroscler..

[38] Morris A.A.M., Spiekerkoetter U., Saudubray J.-M., Baumgartner M.R., Walter J. (2016). Disorders of Mitochondrial Fatty Acid Oxidation & Riboflavin Metabolism. Inborn Metabolic Diseases: Diagnosis and Treatment.

[39] Spiekerkoetter U., Lindner M., Santer R., Grotzke M., Baumgartner M.R., Boehles H., Das A., Haase C., Hennermann J.B., Karall D. (2009). Treatment Recommendations in Long-Chain Fatty Acid Oxidation Defects: Consensus from a Workshop. J. Inherit. Metab. Dis..

[40] Calder P.C. (2011). Fatty Acids and Inflammation: The Cutting Edge between Food and Pharma. Eur. J. Pharmacol..

[41] Radzikowska U., Rinaldi A.O., Çelebi Sözener Z., Karaguzel D., Wojcik M., Cypryk K., Akdis M., Akdis C.A., Sokolowska M. (2019). The Influence of Dietary Fatty Acids on Immune Responses. Nutrients.

[42] El-Gharbawy A., Goldstein A. (2017). Mitochondrial Fatty Acid Oxidation Disorders Associated with Cardiac Disease. Curr. Pathobiol. Rep..

[43] Spiekerkoetter U., Wood P.A. (2010). Mitochondrial Fatty Acid Oxidation Disorders: Pathophysiological Studies in Mouse Models. J. Inherit. Metab. Dis..

[44] Najdekr L., Gardlo A., Mádrová L., Friedecký D., Janečková H., Correa E.S., Goodacre R., Adam T. (2015). Oxidized Phosphatidylcholines Suggest Oxidative Stress in Patients with Medium-Chain Acyl-CoA Dehydrogenase Deficiency. Talanta.

[45] Schuck P.F., Ferreira G.C., Moura A.P., Busanello E.N.B., Tonin A.M., Dutra-Filho C.S., Wajner M. (2009). Medium-Chain Fatty Acids Accumulating in MCAD Deficiency Elicit Lipid and Protein Oxidative Damage and Decrease Non-Enzymatic Antioxidant Defenses in Rat Brain. Neurochem. Int..

[46] Tonin A.M., Grings M., Knebel L.A., Zanatta Â., Moura A.P., Ribeiro C.A.J., Leipnitz G., Wajner M. (2012). Disruption of Redox Homeostasis in Cerebral Cortex of Developing Rats by Acylcarnitines Accumulating in Medium-Chain Acyl-CoA Dehydrogenase Deficiency. Int. J. Dev. Neurosci..

[47] Schuck P.F., Ceolato P.C., Ferreira G.C., Tonin A., Leipnitz G., Dutra-Filho C.S., Latini A., Wajner M. (2007). Oxidative Stress Induction by Cis-4-Decenoic Acid: Relevance for MCAD Deficiency. Free Radic. Res..

[48] Scaini G., Simon K.R., Tonin A.M., Busanello E.N.B., Moura A.P., Ferreira G.C., Wajner M., Streck E.L., Schuck P.F. (2012). Toxicity of Octanoate and Decanoate in Rat Peripheral Tissues: Evidence of Bioenergetic Dysfunction and Oxidative Damage Induction in Liver and Skeletal Muscle. Mol. Cell. Biochem..

[49] Amaral A.U., Cecatto C., da Silva J.C., Wajner A., dos Godoy K.S., Ribeiro R.T., Wajner M. (2016). Cis-4-Decenoic and Decanoic Acids Impair Mitochondrial Energy, Redox and Ca^2+^ Homeostasis and Induce Mitochondrial Permeability Transition Pore Opening in Rat Brain and Liver: Possible Implications for the Pathogenesis of MCAD Deficiency. Biochim. Biophys. Acta (BBA)-Bioenerg..

[50] Reis de Assis D., Maria R.D.C., Borba Rosa R., Schuck P.F., Ribeiro C.A.J., da Costa Ferreira G., Dutra-Filho C.S., Terezinha de Souza Wyse A., Duval Wannmacher C.M., Santos Perry M.L. (2004). Inhibition of Energy Metabolism in Cerebral Cortex of Young Rats by the Medium-Chain Fatty Acids Accumulating in MCAD Deficiency. Brain Res..

[51] Hickmann F.H., Cecatto C., Kleemann D., Monteiro W.O., Castilho R.F., Amaral A.U., Wajner M. (2015). Uncoupling, Metabolic Inhibition and Induction of Mitochondrial Permeability Transition in Rat Liver Mitochondria Caused by the Major Long-Chain Hydroxyl Monocarboxylic Fatty Acids Accumulating in LCHAD Deficiency. Biochim. Biophys. Acta (BBA)-Bioenerg..

[52] Cecatto C., Godoy K.D.S., da Silva J.C., Amaral A.U., Wajner M. (2016). Disturbance of Mitochondrial Functions Provoked by the Major Long-Chain 3-Hydroxylated Fatty Acids Accumulating in MTP and LCHAD Deficiencies in Skeletal Muscle. Toxicol. In Vitro.

[53] Tonin A.M., Ferreira G.C., Grings M., Viegas C.M., Busanello E.N., Amaral A.U., Zanatta Â., Schuck P.F., Wajner M. (2010). Disturbance of Mitochondrial Energy Homeostasis Caused by the Metabolites Accumulating in LCHAD and MTP Deficiencies in Rat Brain. Life Sci..

[54] Cecatto C., Hickmann F.H., Rodrigues M.D.N., Amaral A.U., Wajner M. (2015). Deregulation of Mitochondrial Functions Provoked by Long-Chain Fatty Acid Accumulating in Long-Chain 3-Hydroxyacyl-CoA Dehydrogenase and Mitochondrial Permeability Transition Deficiencies in Rat Heart—Mitochondrial Permeability Transition Pore Opening as a Potential Contributing Pathomechanism of Cardiac Alterations in These Disorders. FEBS J..

[55] Tonin A.M., Amaral A.U., Busanello E.N.B., Grings M., Castilho R.F., Wajner M. (2013). Long-Chain 3-Hydroxy Fatty Acids Accumulating in Long-Chain 3-Hydroxyacyl-CoA Dehydrogenase and Mitochondrial Trifunctional Protein Deficiencies Uncouple Oxidative Phosphorylation in Heart Mitochondria. J. Bioenerg. Biomembr..

[56] Cecatto C., Wajner A., Vargas C.R., Wajner S.M., Amaral A.U., Wajner M. (2018). High Vulnerability of the Heart and Liver to 3-Hydroxypalmitic Acid–Induced Disruption of Mitochondrial Functions in Intact Cell Systems. J. Cell. Biochem..

[57] Tonin A.M., Amaral A.U., Busanello E.N., Gasparotto J., Gelain D.P., Gregersen N., Wajner M. (2014). Mitochondrial Bioenergetics Deregulation Caused by Long-Chain 3-Hydroxy Fatty Acids Accumulating in LCHAD and MTP Deficiencies in Rat Brain: A Possible Role of MPTP Opening as a Pathomechanism in These Disorders?. Biochim. Biophys. Acta (BBA)-Mol. Basis Dis..

[58] Tonin A.M., Grings M., Busanello E.N.B., Moura A.P., Ferreira G.C., Viegas C.M., Fernandes C.G., Schuck P.F., Wajner M. (2010). Long-Chain 3-Hydroxy Fatty Acids Accumulating in LCHAD and MTP Deficiencies Induce Oxidative Stress in Rat Brain. Neurochem. Int..

[59] de Moraes M.S., Guerreiro G., Sitta A., de Moura Coelho D., Manfredini V., Wajner M., Vargas C.R. (2020). Oxidative Damage in Mitochondrial Fatty Acids Oxidation Disorders Patients and the in Vitro Effect of L-Carnitine on DNA Damage Induced by the Accumulated Metabolites. Arch. Biochem. Biophys..

[60] Cecatto C., Amaral A.U., Wajner A., Wajner S.M., Castilho R.F., Wajner M. (2020). Disturbance of Mitochondrial Functions Associated with Permeability Transition Pore Opening Induced by Cis-5-Tetradecenoic and Myristic Acids in Liver of Adolescent Rats. Mitochondrion.

[61] Cecatto C., Amaral A.U., da Silva J.C., Wajner A., Schimit M.D.O.V., da Silva L.H.R., Wajner S.M., Zanatta Â., Castilho R.F., Wajner M. (2018). Metabolite Accumulation in VLCAD Deficiency Markedly Disrupts Mitochondrial Bioenergetics and Ca^2+^ Homeostasis in the Heart. FEBS J..

[62] Cecatto C., Amaral A.U., Roginski A.C., Castilho R.F., Wajner M. (2020). Impairment of Mitochondrial Bioenergetics and Permeability Transition Induction Caused by Major Long-Chain Fatty Acids Accumulating in VLCAD Deficiency in Skeletal Muscle as Potential Pathomechanisms of Myopathy. Toxicol. In Vitro.

[63] Hoffmann L., Seibt A., Herebian D., Spiekerkoetter U. (2014). Monounsaturated 14:1n-9 and 16:1n-9 Fatty Acids but Not 18:1n-9 Induce Apoptosis and Necrosis in Murine HL-1 Cardiomyocytes. Lipids.

[64] McCoin C.S., Knotts T.A., Adams S.H. (2015). Acylcarnitines—Old Actors Auditioning for New Roles in Metabolic Physiology. Nat. Rev. Endocrinol..

[65] Seminotti B., Leipnitz G., Karunanidhi A., Kochersperger C., Roginskaya V.Y., Basu S., Wang Y., Wipf P., Van Houten B., Mohsen A.-W. (2019). Mitochondrial Energetics Is Impaired in Very Long-Chain Acyl-CoA Dehydrogenase Deficiency and Can Be Rescued by Treatment with Mitochondria-Targeted Electron Scavengers. Hum. Mol. Genet..

[66] Hagenbuchner J., Scholl-Buergi S., Karall D., Ausserlechner M.J. (2018). Very Long-/ and Long Chain-3-Hydroxy Acyl CoA Dehydrogenase Deficiency Correlates with Deregulation of the Mitochondrial Fusion/Fission Machinery. Sci. Rep..

[67] Fahy E., Subramaniam S., Brown H.A., Glass C.K., Merrill A.H., Murphy R.C., Raetz C.R.H., Russell D.W., Seyama Y., Shaw W. (2005). A Comprehensive Classification System for Lipids1. J. Lipid Res..

[68] Fahy E., Subramaniam S., Murphy R.C., Nishijima M., Raetz C.R.H., Shimizu T., Spener F., van Meer G., Wakelam M.J.O., Dennis E.A. (2009). Update of the LIPID MAPS Comprehensive Classification System for Lipids1. J. Lipid Res..

[69] Liebisch G., Fahy E., Aoki J., Dennis E.A., Durand T., Ejsing C.S., Fedorova M., Feussner I., Griffiths W.J., Köfeler H. (2020). Update on LIPID MAPS Classification, Nomenclature, and Shorthand Notation for MS-Derived Lipid Structures. J. Lipid Res..

[70] Alves M.A., Lamichhane S., Dickens A., McGlinchey A., Ribeiro H.C., Sen P., Wei F., Hyötyläinen T., Orešič M. (2021). Systems Biology Approaches to Study Lipidomes in Health and Disease. Biochim. Biophys. Acta (BBA)-Mol. Cell Biol. Lipids.

[71] Wei F., Lamichhane S., Orešič M., Hyötyläinen T. (2019). Lipidomes in Health and Disease: Analytical Strategies and Considerations. Trends Anal. Chem..

[72] Martínez G., Jiménez-Sánchez G., Divry P., Vianey-Saban C., Riudor E., Rodés M., Briones P., Ribes A. (1997). Plasma Free Fatty Acids in Mitochondrial Fatty Acid Oxidation Defects. Clin. Chim. Acta.

[73] Costa C.G., Dorland L., Holwerda U., Tavares de Almeida I., Poll-The B.-T., Jakobs C., Duran M. (1998). Simultaneous Analysis of Plasma Free Fatty Acids and Their 3-Hydroxy Analogs in Fatty Acid β-Oxidation Disorders. Clin. Chem..

[74] Kimura M., Yoon H.R., Wasant P., Takahashi Y., Yamaguchi S. (2002). A Sensitive and Simplified Method to Analyze Free Fatty Acids in Children with Mitochondrial Beta Oxidation Disorders Using Gas Chromatography/Mass Spectrometry and Dried Blood Spots. Clin. Chim. Acta.

[75] Onkenhout W., Venizelos V., Scholte H.R., de Klerk J.B.C., Poorthuis B.J.H.M. (2001). Intermediates of Unsaturated Fatty Acid Oxidation Are Incorporated in Triglycerides but Not in Phospholipids in Tissues from Patients with Mitochondrial β-Oxidation Defects. J. Inherit. Metab. Dis..

[76] Zhong S., Li L., Shen X., Li Q., Xu W., Wang X., Tao Y., Yin H. (2019). An Update on Lipid Oxidation and Inflammation in Cardiovascular Diseases. Free Radic. Biol. Med..

[77] Freigang S. (2016). The Regulation of Inflammation by Oxidized Phospholipids. Eur. J. Immunol..

[78] Lund A.M., Dixon M.A., Vreken P., Leonard J.V., Morris A.A.M. (2003). Plasma and Erythrocyte Fatty Acid Concentrations in Long-Chain 3-Hydroxyacyl-CoA Dehydrogenase Deficiency. J. Inherit. Metab. Dis..

[79] Van Hove J.L.K., Kahler S.G., Feezor M.D., Ramakrishna J.P., Hart P., Treem W.R., Shen J.-J., Matern D., Millington D.S. (2000). Acylcarnitines in Plasma and Blood Spots of Patients with Long-Chain 3-Hydroxyacyl-Coenzyme A Dehydrogenase Defiency. J. Inherit. Metab. Dis..

[80] McCoin C.S., Piccolo B.D., Knotts T.A., Matern D., Vockley J., Gillingham M.B., Adams S.H. (2016). Unique Plasma Metabolomic Signatures of Individuals with Inherited Disorders of Long-Chain Fatty Acid Oxidation. J. Inherit. Metab. Dis..

[81] Shen J.J., Matern D., Millington D.S., Hillman S., Feezor M.D., Bennett M.J., Qumsiyeh M., Kahler S.G., Chen Y.-T., Van Hove J.L.K. (2000). Acylcarnitines in Fibroblasts of Patients with Long-Chain 3-Hydroxyacyl-CoA Dehydrogenase Deficiency and Other Fatty Acid Oxidation Disorders. J. Inherit. Metab. Dis..

[82] Alatibi K.I., Hagenbuchner J., Wehbe Z., Karall D., Ausserlechner M.J., Vockley J., Spiekerkoetter U., Grünert S.C., Tucci S. (2021). Different Lipid Signature in Fibroblasts of Long-Chain Fatty Acid Oxidation Disorders. Cells.

[83] Alatibi K.I., Tholen S., Wehbe Z., Hagenbuchner J., Karall D., Ausserlechner M.J., Schilling O., Grünert S.C., Vockley J., Tucci S. (2021). Lipidomic and Proteomic Alterations Induced by Even and Odd Medium-Chain Fatty Acids on Fibroblasts of Long-Chain Fatty Acid Oxidation Disorders. Int. J. Mol. Sci..

[84] van der Veen J.N., Kennelly J.P., Wan S., Vance J.E., Vance D.E., Jacobs R.L. (2017). The Critical Role of Phosphatidylcholine and Phosphatidylethanolamine Metabolism in Health and Disease. Biochim. Biophys. Acta (BBA)-Biomembr..

[85] Li Y., Ge M., Ciani L., Kuriakose G., Westover E.J., Dura M., Covey D.F., Freed J.H., Maxfield F.R., Lytton J. (2004). Enrichment of Endoplasmic Reticulum with Cholesterol Inhibits Sarcoplasmic-Endoplasmic Reticulum Calcium ATPase-2b Activity in Parallel with Increased Order of Membrane Lipids: Implications for Depletion of Endoplasmic Reticulum Calcium Stores and Apoptosis in Cholesterol-Loaded Macrophages. J. Biol. Chem..

[86] Fu S., Yang L., Li P., Hofmann O., Dicker L., Hide W., Lin X., Watkins S.M., Ivanov A., Hotamisligil G.S. (2011). Aberrant Lipid Metabolism Disrupts Calcium Homeostasis Causing Liver Endoplasmic Reticulum Stress in Obesity. Nature.

[87] Braverman N.E., Moser A.B. (2012). Functions of Plasmalogen Lipids in Health and Disease. Biochim. Biophys. Acta (BBA)-Mol. Basis Dis..

[88] Lessig J., Fuchs B. (2009). Plasmalogens in Biological Systems: Their Role in Oxidative Processes in Biological Membranes, Their Contribution to Pathological Processes and Aging and Plasmalogen Analysis. Curr. Med. Chem..

[89] Pujol-Lereis L.M., Liebisch G., Schick T., Lin Y., Grassmann F., Uchida K., Zipfel P.F., Fauser S., Skerka C., Weber B.H.F. (2018). Evaluation of Serum Sphingolipids and the Influence of Genetic Risk Factors in Age-Related Macular Degeneration. PLoS ONE.

[90] Vidaurre O.G., Haines J.D., Katz Sand I., Adula K.P., Huynh J.L., McGraw C.A., Zhang F., Varghese M., Sotirchos E., Bhargava P. (2014). Cerebrospinal Fluid Ceramides from Patients with Multiple Sclerosis Impair Neuronal Bioenergetics. Brain.

[91] Casares D., Escribá P.V., Rosselló C.A. (2019). Membrane Lipid Composition: Effect on Membrane and Organelle Structure, Function and Compartmentalization and Therapeutic Avenues. Int. J. Mol. Sci..

[92] van Meer G., Voelker D.R., Feigenson G.W. (2008). Membrane Lipids: Where They Are and How They Behave. Nat. Rev. Mol. Cell Biol..

[93] Paradies G., Paradies V., Ruggiero F.M., Petrosillo G. (2019). Role of Cardiolipin in Mitochondrial Function and Dynamics in Health and Disease: Molecular and Pharmacological Aspects. Cells.

[94] Kreutzberger A.J.B., Ji M., Aaron J., Mihaljević L., Urban S. (2019). Rhomboid Distorts Lipids to Break the Viscosity-Imposed Speed Limit of Membrane Diffusion. Science.

[95] Sezgin E., Gutmann T., Buhl T., Dirkx R., Grzybek M., Coskun Ü., Solimena M., Simons K., Levental I., Schwille P. (2015). Adaptive Lipid Packing and Bioactivity in Membrane Domains. PLoS ONE.

[96] Tan S.T., Ramesh T., Toh X.R., Nguyen L.N. (2020). Emerging Roles of Lysophospholipids in Health and Disease. Prog. Lipid Res..

[97] Tucci S., Behringer S., Spiekerkoetter U. (2015). De Novo Fatty Acid Biosynthesis and Elongation in Very Long-Chain Acyl-CoA Dehydrogenase-Deficient Mice Supplemented with Odd or Even Medium-Chain Fatty Acids. FEBS J..

[98] Tucci S., Flögel U., Spiekerkoetter U. (2015). Sexual Dimorphism of Lipid Metabolism in Very Long-Chain Acyl-CoA Dehydrogenase Deficient (VLCAD−/−) Mice in Response to Medium-Chain Triglycerides (MCT). Biochim. Biophys. Acta (BBA)-Mol. Basis Dis..

[99] Sklirou E., Alodaib A.N., Dobrowolski S.F., Mohsen A.-W.A., Vockley J. (2021). Physiological Perspectives on the Use of Triheptanoin as Anaplerotic Therapy for Long Chain Fatty Acid Oxidation Disorders. Front. Genet..

[100] Tucci S. (2017). Very Long-Chain Acyl-CoA Dehydrogenase (VLCAD-) Deficiency–Studies on Treatment Effects and Long-Term Outcomes in Mouse Models. J. Inherit. Metab. Dis..

[101] Alatibi K.I., Wehbe Z., Spiekerkoetter U., Tucci S. (2020). Sex-Specific Perturbation of Complex Lipids in Response to Medium-Chain Fatty Acids in Very Long-Chain Acyl-CoA Dehydrogenase Deficiency. FEBS J..

[102] Ntambi J.M. (1995). The Regulation of Stearoyl-CoA Desaturase (SCD). Prog. Lipid Res..

[103] Opalka J.R., Gellerich F.-N., Zierz S. (2001). Age and Sex Dependency of Carnitine Concentration in Human Serum and Skeletal Muscle. Clin. Chem..

[104] Wehbe Z., Alatibi K., Jellusova J., Spiekerkoetter U., Tucci S. (2019). The Fate of Medium-Chain Fatty Acids in Very Long-Chain Acyl-CoA Dehydrogenase Deficiency (VLCADD): A Matter of Sex?. Biochim. Biophys. Acta (BBA)-Mol. Cell Biol. Lipids.

[105] Otsuka M., Tsuchiya S., Aramaki Y. (2004). Involvement of ERK, a MAP Kinase, in the Production of TGF-β by Macrophages Treated with Liposomes Composed of Phosphatidylserine. Biochem. Biophys. Res. Commun..

[106] Fadok V.A., de Cathelineau A., Daleke D.L., Henson P.M., Bratton D.L. (2001). Loss of Phospholipid Asymmetry and Surface Exposure of Phosphatidylserine Is Required for Phagocytosis of Apoptotic Cells by Macrophages and Fibroblasts. J. Biol. Chem..

[107] Di Paolo G., De Camilli P. (2006). Phosphoinositides in Cell Regulation and Membrane Dynamics. Nature.

