# Supplementary material for: Mitochondrial Fatty Acid β-Oxidation Disorders: From Disease to Lipidomic Studies—A Critical Review
Source: Int J Mol Sci. 2022 Nov 11;23(22):13933. doi: 10.3390/ijms232213933 (PMC9696092; doi:10.3390/ijms232213933)
Supplement: Supplementary file 1 [file ijms-23-13933-s001.zip › ijms-1943445-supplementary.pdf]

Review

Mitochondrial Fatty Acid β-Oxidation Disorders: From Disease to Lipidomics Studies - A Critical Review

Inês M. S. Guerra[1,2], Helena B. Ferreira[1,2], Tânia Melo[1,2], Hugo Rocha[3,4], Sónia Moreira[5,6], Luísa Diogo[6], M. Rosário Domingues[1,2], Ana S. P. Moreira[1,*]

[1] Mass Spectrometry Center, LAQV-REQUIMTE, Department of Chemistry, University of Aveiro, Campus Universitário de Santiago, 3810-193, Aveiro, Portugal
[2] Centre for Environmental and Marine Studies - CESAM, Department of Chemistry, University of Aveiro, Campus Universitário de Santiago, 3810-193 Aveiro, Portugal
[3] Newborn Screening, Metabolism and Genetics Unit, Human Genetics Department, National Institute of Health Doutor Ricardo Jorge, 4000-053 Porto, Portugal
[4] Department of Pathological, Cytological and Thanatological Anatomy, School of Health, Polytechnic Institute of Porto, Porto, Portugal
[5] Internal Medicine, Centro Hospitalar e Universitário de Coimbra, 3000-075, Coimbra, Portugal
[6] Reference Center of Inherited Metabolic Diseases, Hospital Pediátrico, Centro Hospitalar e Universitário de Coimbra, 3000-075, Coimbra, Portugal
* Correspondence: ana.moreira@ua.pt

Supplementary Material

Supplementary Table S1: Additional information of the published studies on lipidomics in FAOD.

Analytical Method	Lipid Extraction Method	Type of Sample	Type of FAOD	Number of patients	Age Range	Reference
GC-MS Methylation of nonesterified FA(acetyl chloride/metanol 50:1 (v/v))	No extraction step	Plasma	MCADD	6	INF	Martínez et al. [1]
			LCHADD	3	INF	
			VLCADD	4	INF	
GC-MS simultaneous trifluoroacetylation (TFA) of hydroxyl groups and tert-butyldimethylsilylation (tBDMS) of the carboxyl groups	ethyl acetate	Plasma	LCHADD/MTP	5/1	INF	Costa et al. [2]
			VLCADD	2	INF	
			MCADD	1	INF	
			CPT-I/CPT-II	1/1	INF	
GC-FID FAMES (methanol/benzene 4:1 (v/v) and acetyl chloride, 100ºC, 1h)	Chloroform/Methanol (2:1, V/V)	Post mortem liver tissue	MCADD	1 (F)	2.2 yr	Onkenhout et al. [3]
		Post mortem liver and muscle tissue	VLCADD	2 (M)	2 mth	

GC-FID FAMES (methanol/benzene 4:1 (v/v) and acetyl chloride, 80ºC, 4h)	No extraction step	Plasma	LCHADD	9	3.5-10.6 yr	Lund et al. [4]
		RBC				
GC-MS Methylation of nonesterified FA (acetyl chloride/metanol 50:1 (v/v))	Acetyl chloride + 6% potassium carbonate + hexane	DBS	MCADD	2	INF	Kimura et al. [5]
			VLACDD	4	INF	
LSI-MS	INF	Plasma	LCHADD	23 (9M+14F)	INF	van Hove et al. [6]
		DBS				
Triple Quadupole-MS/MS	Hexane	Human skin fibroblats	LCHADD	5	INF	Shen et al. [7]
ESI-MS/MS	Acetonitrile/water (80/20, v/v)	DBS from VLCAD-/- mice	VLCAD	M and F	6 mth	Tucci et al. [8]
GC-MS FAMEs (trimethylsulfonium hydroxide)	Chloroform/Methanol (1:2, V/V)	Hepatic and cardiac tissue from VLCAD-/-mice	VLCADD	INF	5-7 w	Tucci et al. [9]
UHLC-MS/MS base Metabolomics	INF	Plasma	VLCADD	8	INF	Sklirou et al. [10]

Shotgun lipidomics ESI-Qexactive-MS and MS/MS	Chloroform/Methanol (17:1, V/V)	Murine fibroblats from WT and VLCAD-/-mice	VLCADD	M and F	9-10 w	Alatibi et al. [11]
Shotgun lipidomics ESI-Qexactive-MS and MS/MS	Chloroform/Methanol (17:1, V/V)	Human fibroblast	VLCADD	6 (3M+3F)	INF	Alatibi et al. [12]
			LCHADD	3(2M+1F)	INF	
			CPT-II	2(1M+1F)	INF	
Shotgun lipidomics ESI-Qexactive-MS and MS/MS	Chloroform/Methanol (17:1, V/V)	Human fibroblast	VLCADD	6 (3M+3F)	INF	Alatibi et al. [13]
			LCHADD	3(2M+1F)	INF	
CSH-ESI QTOF MS/MS	MTBE	Plasma	LCHADD and CPT-II	12 (7M+5F)	14.7 yr	McCoin et al. [14]
LC-MS based untarget metabolomics using amino-propyl colunm	methanol/water	DBS	MCADD	8	INF	Najderkr et al. [15]

References

1. Martínez, G.; Jiménez-Sánchez, G.; Divry, P.; Vianey-Saban, C.; Riudor, E.; Rodés, M.; Briones, P.; Ribes, A. Plasma Free Fatty Acids in Mitochondrial Fatty Acid Oxidation Defects. *Clinica Chimica Acta* **1997**, *267*, 143–154, doi:10.1016/S0009-8981(97)00130-7.
2. Costa, C.G.; Dorland, L.; Holwerda, U.; Tavares de Almeida, I.; Poll-The, B.-T.; Jakobs, C.; Duran, M. Simultaneous Analysis of Plasma Free Fatty Acids and Their 3-Hydroxy Analogs in Fatty Acid β-Oxidation Disorders. *Clinical Chemistry* **1998**, *44*, 463–471, doi:10.1093/clinchem/44.3.463.
3. Onkenhout, W.; Venizelos, V.; Scholte, H.R.; de Klerk, J.B.C.; Poorthuis, B.J.H.M. Intermediates of Unsaturated Fatty Acid Oxidation Are Incorporated in Triglycerides but Not in Phospholipids in Tissues from Patients with Mitochondrial β-Oxidation Defects. *Journal of Inherited Metabolic Disease* **2001**, *24*, 337–344, doi:10.1023/A:1010592232317.
4. Lund, A.M.; Dixon, M.A.; Vreken, P.; Leonard, J.V.; Morris, A. a. M. Plasma and Erythrocyte Fatty Acid Concentrations in Long-Chain 3-Hydroxyacyl-CoA Dehydrogenase Deficiency. *Journal of Inherited Metabolic Disease* **2003**, *26*, 410–412, doi:10.1023/A:1025175606891.

5. Kimura, M.; Yoon, H.R.; Wasant, P.; Takahashi, Y.; Yamaguchi, S. A Sensitive and Simplified Method to Analyze Free Fatty Acids in Children with Mitochondrial Beta Oxidation Disorders Using Gas Chromatography/Mass Spectrometry and Dried Blood Spots. *Clinica Chimica Acta* **2002**, *316*, 117–121, doi:10.1016/S0009-8981(01)00741-0.
6. Van Hove, J.L.K.; Kahler, S.G.; Feezor, M.D.; Ramakrishna, J.P.; Hart, P.; Treem, W.R.; Shen, J.-J.; Matern, D.; Millington, D.S. Acylcarnitines in Plasma and Blood Spots of Patients with Long-Chain 3-Hydroxyacyl-Coenzyme A Dehydrogenase Defiency. *Journal of Inherited Metabolic Disease* **2000**, *23*, 571–582, doi:10.1023/A:1005673828469.
7. Shen, J.J.; Matern, D.; Millington, D.S.; Hillman, S.; Feezor, M.D.; Bennett, M.J.; Qumsiyeh, M.; Kahler, S.G.; Chen, Y.-T.; Van Hove, J.L.K. Acylcarnitines in Fibroblasts of Patients with Long-Chain 3-Hydroxyacyl-CoA Dehydrogenase Deficiency and Other Fatty Acid Oxidation Disorders. *Journal of Inherited Metabolic Disease* **2000**, *23*, 27–44, doi:10.1023/A:1005694712583.
8. Tucci, S.; Flögel, U.; Spiekerkoetter, U. Sexual Dimorphism of Lipid Metabolism in Very Long-Chain Acyl-CoA Dehydrogenase Deficient (VLCAD−/−) Mice in Response to Medium-Chain Triglycerides (MCT). *Biochimica et Biophysica Acta (BBA) - Molecular Basis of Disease* **2015**, *1852*, 1442–1450, doi:10.1016/j.bbadis.2015.04.009.
9. Tucci, S.; Behringer, S.; Spiekerkoetter, U. De Novo Fatty Acid Biosynthesis and Elongation in Very Long-Chain Acyl-CoA Dehydrogenase-Deficient Mice Supplemented with Odd or Even Medium-Chain Fatty Acids. *The FEBS Journal* **2015**, *282*, 4242–4253, doi:https://doi.org/10.1111/febs.13418.
10. Sklirou, E.; Alodaib, A.N.; Dobrowolski, S.F.; Mohsen, A.-W.A.; Vockley, J. Physiological Perspectives on the Use of Triheptanoin as Anaplerotic Therapy for Long Chain Fatty Acid Oxidation Disorders. *Frontiers in Genetics* **2021**, *11*.
11. Alatibi, K.I.; Wehbe, Z.; Spiekerkoetter, U.; Tucci, S. Sex-Specific Perturbation of Complex Lipids in Response to Medium-Chain Fatty Acids in Very Long-Chain Acyl-CoA Dehydrogenase Deficiency. *The FEBS Journal* **2020**, *287*, 3511–3525, doi:10.1111/febs.15221.
12. Alatibi, K.I.; Hagenbuchner, J.; Wehbe, Z.; Karall, D.; Ausserlechner, M.J.; Vockley, J.; Spiekerkoetter, U.; Grünert, S.C.; Tucci, S. Different Lipid Signature in Fibroblasts of Long-Chain Fatty Acid Oxidation Disorders. *Cells* **2021**, *10*, 1239, doi:10.3390/cells10051239.
13. Alatibi, K.I.; Tholen, S.; Wehbe, Z.; Hagenbuchner, J.; Karall, D.; Ausserlechner, M.J.; Schilling, O.; Grünert, S.C.; Vockley, J.; Tucci, S. Lipidomic and Proteomic Alterations Induced by Even and Odd Medium-Chain Fatty Acids on Fibroblasts of Long-Chain Fatty Acid Oxidation Disorders. *International Journal of Molecular Sciences* **2021**, *22*, 10556, doi:10.3390/ijms221910556.
14. McCoin, C.S.; Piccolo, B.D.; Knotts, T.A.; Matern, D.; Vockley, J.; Gillingham, M.B.; Adams, S.H. Unique Plasma Metabolomic Signatures of Individuals with Inherited Disorders of Long-Chain Fatty Acid Oxidation. *Journal of Inherited Metabolic Disease* **2016**, *39*, 399–408, doi:10.1007/s10545-016-9915-3.
15. Najdekr, L.; Gardlo, A.; Mádrová, L.; Friedecký, D.; Janečková, H.; Correa, E.S.; Goodacre, R.; Adam, T. Oxidized Phosphatidylcholines Suggest Oxidative Stress in Patients with Medium-Chain Acyl-CoA Dehydrogenase Deficiency. *Talanta* **2015**, *139*, 62–66, doi:10.1016/j.talanta.2015.02.041.
